# Design, spectroscopic analysis, DFT calculations, adsorption evaluation, molecular docking, comprehensive in silico and in vitro bioactivity studies of thiocarbohydrazide grafted dialdehyde cellulose nanobiosorbent

**DOI:** 10.1038/s41598-025-96525-2

**Published:** 2025-04-17

**Authors:** Magda A. Akl, Aya G. Mostafa, Abdelrahman S. El-Zeny, El-Sayed R. H. El-Gharkawy

**Affiliations:** Department of Chemistry, Faculty of Science, Mansoura University, Mansoura, 35516 Egypt

**Keywords:** Thiocarbohydrazide, Adsorption, DFT, In silico toxicity, Molecular docking, Antibacterial, Ag^+^, Hg^2+^, Cu^2+^, Analytical chemistry, Environmental chemistry, Materials chemistry, Physical chemistry, Theoretical chemistry

## Abstract

Heavy metals have attracted considerable attention lately because of their widespread occurrence in aquatic environments and potential biological toxicity to animals and human. The current investigation focused on synthesizing the DAC@TCH nanobiosorbent by coupling dialdehyde cellulose with thiocarbohydrazide ligand. Subsequent characterization of DAC@TCH was carried out utilizing various analytical methods such as elemental analysis, scanning electron microscopy (SEM), transmission electron microscopy (TEM), Fourier transform infrared (FT-IR), and thermogravimetric analysis (TGA and DTA). DFT calculations were utilized to verify the molecular structure, analysis of frontier molecular orbitals (FMOs), molecular electrostatic potential (MEP) and reactivity descriptor for all phases. In vitro experiments were conducted to evaluate the biological properties of the DAC@TCH nanobiosorbent. These findings revealed that the synthesized DAC@TCH nanobiosorbent has been observed to show effective antibacterial IZD value against *E. Coli* (28 mm) which is superior to the efficacy of standard drug amoxicillin used (5 mm). Furthermore, in silico antibacterial activities (molecular docking) of the DAC@TCH have indicated this to exhibit excellent efficacy with docking score of (**−**7.4237 kcal/mol) and (−7.1325 kcal/mol) for *S. aureus and, E. coli,* respectively. Meanwhile the binding energies (best docking scores) in kcal/mol for Amoxicillin are (−5.8090) and (−6.7442) for *S. aureus and, E. coli,* respectively. Drug-likeness rules like Lipinski’s, Veber’s and Egan’s were considered for a more comprehensive evaluation. The prepared DAC@TCH nanobiosorbent was investigated for its potential to adsorb metal ions (Ag^+^, Hg^2+^, and Cu^2+^) from diverse water samples. Optimal conditions including pH, temperature, DAC@TCH dosage, oscillation time, initial metal ion concentration, and interference from other ions were explored. The adsorption of Hg^2+^, Cu^2+^, and Ag^+^ ions by DAC@TCH followed pseudo-second-order kinetics and Langmuir isothermal model, achieving maximum adsorption capacities of 196 mg/g for Ag^+^, 190 mg/g for Hg^2+^, and 73 mg/g for Cu^2+^. The adsorption process was determined to be exothermic and spontaneous across varying temperatures. Additionally, over 95% of adsorbed metal ions were effectively desorbed using thiourea (5%) and 0.3 M HNO_3_ elution mixture. DAC@TCH nanobiosorbent demonstrated excellent reusability, retaining its adsorption capacity through five cycles without degradation. The study highlights the potential of DAC@TCH for efficient recovery of heavy metals from different water sources, considering its application versatility, reusability, and minimal interference. Furthermore, the plausible mechanism of Ag^+^, Hg^2+^, and Cu^2+^ adsorption onto DAC@TCH bionanosorbent is elucidated.

## Introduction

The escalation of heavy metal environmental pollution poses a significant global challenge, drawing increasing concern due to its detrimental impacts. These inorganic contaminants are being released into our water bodies, soils, and atmosphere, primarily stemming from the expanding agricultural and metal industries, alongside inadequate waste management practices and the widespread use of fertilizers and pesticides^1^.

In recent decades, the issue of water contamination has escalated due to population growth and the consequent rise in wastewater production, leading to a pressing concern. Owing to heavy metals’ persistence in the ecosystem, they have emerged as prominent pollutants in wastewater. Consequently, there is a concerted effort towards researching methods and technologies for the treatment and recovery of heavy metals from wastewater^2–5^. Heavy metal pollution primarily stems from industrial activities, including metal processing, fertilizer production, paint manufacturing, electroplating, battery production, and printing processes. Additionally, natural processes such as weathering, volcanic eruptions, and the degradation of bedrock containing toxic metal ions contribute to heavy metal contamination in the environment^6^.

Various techniques are available for separating and removing heavy metals from different samples, including solvent extraction, solid-phase extraction, flotation, ion exchange, and precipitation. Among these methods, solid-phase extraction (SPE) stands out due to several advantages. SPE is known for its ease of use, rapidity, reduced reliance on organic solvents, versatility in adapting to different determination methods, and high enrichment factor for heavy metal recovery^7–12^.

Adsorption is a method that garners significant scientific interest due to its effectiveness, affordability, ease of use, and the wide availability of diverse adsorbents. Researchers have directed their efforts toward finding low-cost and easily accessible materials for wastewater treatment. Natural polymers, in particular, have gained considerable attention for their suitability in water treatment. Well-known examples include chitosan, alginate, lignin, and cellulose^13–16^.

Cellulose is the most abundant natural polymer. In addition, cellulose is considered one of the most promising eco-friendly, biodegradable, and renewable resources^17^. Native cellulose typically lacks the necessary selectivity or adsorption capacity for metal ions because it lacks active binding sites crucial for the adsorption process. To enhance its selectivity and effectiveness in adsorbing various metal ions, a range of chemical and physical approaches have been employed to modify cellulose^18^. Cellulose can readily undergo modification by introducing functional groups through reactions with its hydroxyl groups. This modification process enhances cellulose’s ability to selectively adsorb various metal ions with improved adsorption capacity. For instance, incorporating sulfonic acid groups onto cellulose has been shown to significantly enhance its selectivity and adsorption capacity for different metal ions^19^, succinyl groups^20,21^, amino groups^22^, and carboxyl groups^23^. Furthermore, heavy metals tend to strongly coordinate with nitrogen (N) and sulfur (S)-donor ligands, aligning with Pearson’s hard and soft acid–base concept^24^.

Many materials were applied for the heavy metals removal and exhibited high affinities such as activated carbon, polymer & polymer-based composites, zeolites, agricultural solid waste-based biocomposites, clay minerals, and biomass-based materials^25–28^. Several cellulose-based adsorbents have been reported to be utilized for the remediation of heavy metals. (Zhu et al.) synthesized the cellulose II-based spherical nanoparticle micro cluster material and utilized it for the Cr(VI) adsorption^29^. (Akl et al.) utilized the di-aminoguanidine functionalized cellulose for the Hg(II), Cd(II), Cu(II), and Pb(II) removal^30^. Moreover, the removal of Cu^2+^, Hg^2+^, and Pb^2+^ was investigated using guanyl thiosemicarbazide functionalized dialdehyde cellulose^31^. (Mostafa et al.) utilized aminothiol-supported cellulose composite for Hg(II) remediation^32^.

Molecular docking emerges as a crucial tool in comprehending the interactions between molecules and proteins at the atomic level. By arranging ligands in suitable orientations and conformations, molecular docking emphasises the biological reactions that take place between the two components. This approach is particularly vital in assessing the binding affinity between receptor proteins in biological systems and ligand molecules, a pivotal aspect in drug discovery and delivery endeavours.

Thiocarbohydrazides represent a vital class of compounds with diverse applications across various fields. Their chemistry has garnered significant interest in both synthetic organic chemistry and biological research. Thiocarbohydrazides find extensive utility in several applications, including the evaluation of interphase nuclei, analysis of tissue three-dimensional ultrastructure, and therapeutic interventions^33^.

According to our knowledge, dialdehyde cellulose modification using a thiocarbohydrazide has not been reported in the literature. Furthermore, no data were found on utilizing DAC@TCH modified cellulose as an effective adsorbent for Cu^2+^, Hg^2+^, and Ag^+^ removal from real polluted water samples.

The present investigation aims to achieve several objectives: (i) Preparation of modified cellulosic material (DAC@TCH) enriched with nitrogen (N) and sulfur (S) active groups to facilitate adsorption of Ag^+^, Hg^2+^, and Cu^2+^ metal ions, both individually and in multi-component solutions; (ii) Characterization of DAC@TCH through various analytical techniques including optical imaging, elemental analysis, (FTIR), (SEM), and (TEM); (iii) Investigation of Ag^+^, Hg^2+^, and Cu^2+^ adsorption by DAC@TCH through batch sorption experiments; (iv) Determination of optimum adsorption parameters such as pH, temperature, initial concentrations of the three metal ions, DAC@TCH dosage, agitation time, and interference from other ions; (v) Study of Ag^+^, Hg^2+^, and Cu^2+^ adsorption kinetics, isotherms, and thermodynamics; (vi) Evaluation and confirmation of the frontier molecular orbitals (LUMO and HOMO), dipole moment (µ), energy gap, and structure-activity relationship of cellulose using Density Functional Theory (DFT); (v). Molecular docking, comprehensive in silico and in vitro bioactivity studies of DAC@TCH; (vi) Comparison of the efficiency of DAC@TCH with other previously reported adsorbents for adsorption of Ag^+^, Hg^2+^, and Cu^2+^; (vii) Explanation of the adsorption mechanism of Ag^+^, Hg^2+^, and Cu^2+^ onto DAC@TCH.

## Experimental

### Materials

All the chemicals and reagents that were used in this work were pure analytical reagent grade. The water utilized throughout the experimental work was double distilled water (DDW). Thiocarbohydrazide (TCH, 98%), Sodium hydroxide (NaOH, 99,99%), micro granular cellulose powder, Potassium periodate (KIO_4_), Mercuric chloride (HgCl_2_), Cupper chloride (CuCl_2_), and Silver nitrate (AgNO_3_) were purchased from Sigma-Aldrich. Nitric acid (HNO_3_, 65%), Hydrochloric acid (HCl, 37%), Sodium acetate dihydrate, and glacial acetic acid were bought from Merck Company, Germany. To prepare metal stock solutions (1000 mg/L), the appropriate amounts of HgCl_2_, CuCl_2_, and AgNO_3_ metal salts were dissolved in one liter of DDW.

### Preparations

#### Preparation of 2, 3 dialdehyde cellulosic (DAC)

One gram of native cellulose powder was immersed in 0.2 L of KIO_4_ (3 g/L) and the pH of the periodate solution was adjusted to 5 by using acetate buffer. In complete absence of the light, the previous mixture was stirred for two hours at 45^ο^C. Then, it was mixed with 0.2 L of ethylene glycol solution (1%) and stirred for 30 min to stop the oxidation process. Finally, the mixture was filtered, washed numerous times using ethanol (99.5%), and dried in the oven at about 40^ο^C^34^.

##### Determination of aldehyde content

Based on previously published studies, the aldehyde content for DAC was estimated^35–37^.

The aldehyde content for DAC was calculated as shown in Eq. (1):

1 $$AC\left(\%\right)=\frac{{M}_{NaOH}({V}_{sample}-{V}_{control})}{\raisebox{1ex}{$m$}\!\left/ \!\raisebox{-1ex}{$Mwt$}\right.}\times 100$$

where M_NaOH_ is the used NaOH concentration (0.1 mol), m is the prepared DAC weight and Mwt is the molecular weight of the DAC repeating unit (C_6_H_8_O_10_)_n_ that is equal to 160.124 g/mol. V_sample_ and V_control_ are the recorded volumes of the NaOH consumption for the DAC sample and control one, respectively.

#### Preparation of DAC@TCH

**Fig. 1:**
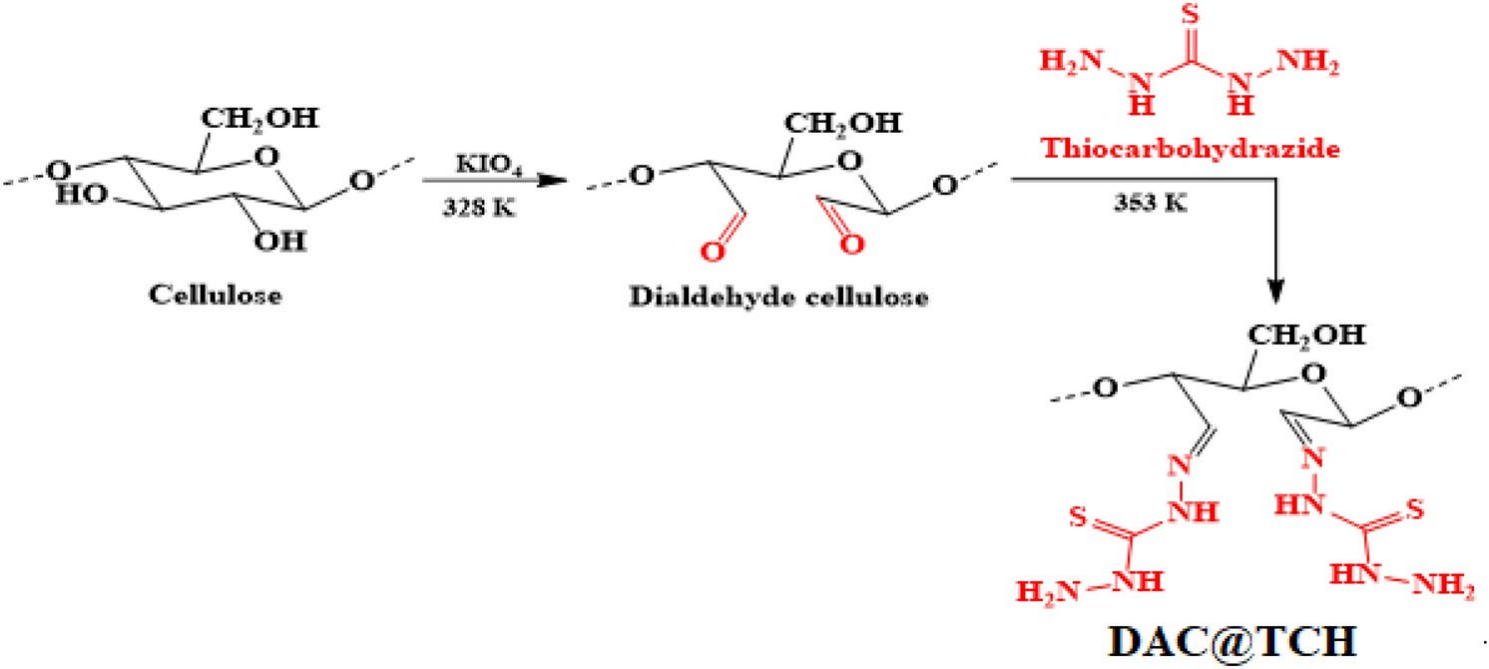
Synthesis of DAC@TCH.

The preparation of DAC@TCH adsorbent was carried out by refluxing 1 g of DAC with 0.2 L of 1% w/v alcoholic thiocarbohydrazide solution at 353 K for 5 h with adding 3 drops of triethylamine (TEA). To remove any excess unreacted thiocarbohydrazide after the refluxing process, the mixture was filtered and then washed several times with ethanol (99.95%) followed by washing with hot DDW. Finally, the prepared DAC@TCH is oven-dried at 323 K. The synthesis mechanism of DAC@TCH is presented in (Fig. 1).

### Characterization of samples

Elemental analysis (EA) of both native and DAC@TCH samples was performed using a Perkin-Elmer 2400 CHNS analyzer. Fourier transform infrared (FT-IR) spectra of native cellulose, DAC, and DAC@TCH before and after adsorption were obtained using a Shimadzu 5800 FT-IR spectrometer, with samples prepared as KBr-pressed discs. Surface morphology of native cellulose, DAC and DAC@TCH before and after adsorption of Cu^2+^ ions were studied after sputter-coating with Au ions by using the scanning electron microscope (Quanta FEG-250). Transmission Electron Microscopy (H-7650 Hitachi, Japan) was used to determine DAC@TCH size and pore size distribution. TGA and DTG were recorded using a thermo analyzer Shimatzu DT40 (Japan) in the temperature range of 30–800 °C. The concentration of Cu^2+^, Hg^2+^, and Ag^+^ was determined using Agilent’s 5100 ICP OES instrument.

### Adsorption and desorption experiments

Adsorption–desorption of heavy metals (Cu^2+^, Hg^2+^, and Ag^+^) from polluted water was carried out by inserting 0.05 g of DAC@TCH in 5 ml of polluted water with varying shaking time, metal initial concentration, and pH. The investigated water samples were treated through acidification using HNO_3_ (2%) prior to the adsorption–desorption investigation. The concentrations of Cu^2+^, Hg^2+^, and Ag^+^ after the adsorption process were determined using ICP OES. The adsorption efficiency (q_e_), removal (R%), the capacity of the desorption (q_d_), and desorption (D%) were estimated from the following Eq. (2), Eq. (3), Eq. (4), and Eq. (5), respectively^38^.

2 $${\text{q}}_{e}=\frac{({\text{C}}_{i}-{\text{C}}_{e})\text{V}}{m}$$

3 $$\text{R }(\text{\%})=\frac{({\text{C}}_{i}-{\text{C}}_{e})}{{\text{C}}_{i}}\times 100$$

4 $${\text{q}}_{d} =\frac{{\text{C}}_{d}\text{ V}}{m}$$

5 $${{D}}_{e} (\text{\%})=\frac{{\text{q}}_{d}}{{q}_{e}}\times 100$$

Herein, q_e_ (mg/g) is the DAC@TCH adsorption capacity. C_i_ (mg/L) and C_e_ (mg/L) are the initial metal ion concentrations and the equilibrium concentration after adsorption, respectively. While V (L) is the volume of metal ions solution. m is the mass of the DAC@TCH adsorbent (g), q_d_ is the desorption capacity in (mg/g), C_d_ is the concentration of metal ions in the used eluent (mg/L) after the desorption experiment, and De (%) is the desorption efficiency.

#### Batch tests

Batch tests were performed to achieve the optimum adsorption conditions. These investigations were carried out using 0.05 g of DAC@TCH (except for the adsorbent dose effect investigation, mass was varied between 0.01 g to 0.1 g) added to 50 ml of 100 mg/L of (Cu^2+^, Hg^2+^, and Ag^+^). The pH was extended from 1 to 9. For Cu^2+^ and Hg^2+^, the solution pH was adjusted using 0.1 M NaOH and/or 0.1 M HCL. While the Ag^+^ solution pH was adjusted by acetate buffer.

The temperature effect was investigated in the range of (20–45 °C). Initial (Cu^2+^, Hg^2+^, and Ag^+^) concentrations ranged between 25–300 mg/L. Oscillation time was first set to 4 h and then extended between 15–240 min to evaluate the optimum contact time. Concentrations of Cu^2+^, Hg^2+^, and Ag^+^ ions in each experiment were measured by ICP OES. Then, the DAC@TCH adsorption capacity (q_e_) and the Cu^2+^, Hg^2+^, and Ag^+^ ions removal efficiency (R (%)) were calculated as Eq. (2) and Eq. (3), respectively.

### Biological activity studies of DAC@TCH

The antibacterial activity of DAC@TCH was tested against two types of bacteria such as Gram-positive bacteria (*S. aureus*) and Gram-negative bacteria (*E. coli*) by Agar well diffusion method. Agar well diffusion method is widely used to evaluate the antimicrobial activity of plants or microbial extracts. Similarly to the procedure used in disk-diffusion method, the agar plate surface is inoculated by spreading a volume of the microbial inoculum over the entire agar surface. Then, a hole with a diameter of 9 mm is punched aseptically with a sterile cork borer or a tip, and a volume (100 µL) of the antimicrobial agent which suspended in two type of solvent (water and, Dmso) in desired concentration (35 mg/ml) is introduced into the well. The samples were seeded in Petri dishes containing agar media and the Petri dishes were incubated for 5 h at 36 °C. The antibacterial activity was recorded by measuring the diameter zone of inhibition after 24 h of incubation and summarized in (Table 2) Obtained data was also compared with standard Amoxicillin drug to calculate the activity index at room temperature. The antimicrobial activity percent (%) was calculated according to Eq. (6) ^39,40^.

6 $$\% Activity \left( A \right) = \left( {\frac{{zone of inhibition by test compound \left( {diameter } \right)}}{{zone of inhibition by standard \left( {diameter } \right)}}} \right) \times 100$$

**Table 1:** Global reactivity descriptors (GRD).

GRD	Equation
The energy gap (ΔE_gap_)	ΔE_gap_ = (E_LUMO_ − E_HOMO_)
Ionization potential (I_P_)	I_P_ = −E_HOMO_
Electron affinity (E_A_)	E_A_ = −E_LUMO_
Hardness (η)	$\eta =\frac{{I}_{P}-{E}_{A}}{2}$
Softness (σ)	$\sigma =\frac{1}{\eta }$
Electronegativity (χ)	$\text{\rm X}=\frac{{I}_{P}+{E}_{A}}{2}$
Chemical potentials (µ)	µ = −**χ**
The fraction of electron transferred (ΔN)	$\left(\text{N}\right)=\frac{{X}_{Ce}-{X}_{Ligand}}{2({\eta }_{Ce}+{X}_{Ligand})}$
Electrophilicity index (ω)	$\left({\varvec{\omega}}\right)=\frac{{{\varvec{\mu}}}^{2}}{2{\varvec{\eta}}}$
The back donation^54^	$\left(\text{Eback}-\text{donation}\right)=-\frac{\eta }{4}$

### DFT calculations

Geometry optimizations and other density functional theory (DFT) calculations were conducted on native cellulose, DAC, and DAC@TCH. DFT is recognized as a cost-effective approach to approximate electron correlation effects. All DFT calculations were performed using the B3LYP level of theory, which combines Becke’s three-parameter (B3) exchange functional with the Lee–Yang–Parr (LYP) correlation functional^41^. The B3LYP level is widely utilized for studying organic electronic compounds due to its ability to reliably predict geometries and provide accurate estimations of HOMO–LUMO (Highest Occupied Molecular Orbital—Lowest Unoccupied Molecular Orbital) gaps, which are in good agreement with experimental values^42–44^. All geometry optimization calculations were performed using the B3LYP/6-31 g(d) level of theory for all atoms. These computations were conducted utilizing the Gaussian09 suite of programs^45^. Gauss View 5.0 package^46^ was used to obtain various graphic views of molecular shapes of distinctive molecular orbitals.

Highest occupied molecular orbitals (HOMO) and lowest unoccupied molecular orbitals (LUMO) are very considerable elements of theoretical molecular design. The electronic properties and reactivity definers [such as ionization potential (I_P_), electron affinity (E_A_), hardness ($\eta )$, softness ($\sigma$), and electronegativity ($\chi$)] can be determined from the HOMO and LUMO orbital energies through Koopman’s theorem^47^.

The global reactivity descriptors (GRD) are presented in (Table 1); Ionization potential (I_P_), electron affinity (E_A_), electronegativity ($\chi$), global hardness ($\eta )$ and softness ($\sigma$), can be explained in terms of the energy of the HOMO and the LUMO^48–53^.

### Molecular docking

Scientists working on drug design and discovery can benefit from theoretical computations such as molecular docking, which can be used to propose drug interaction models and provide details on how novel drugs behave towards biological targets. The Protein Data Bank structure of two types of bacteria such as Gram-positive bacteria (*Staphylococcus aureus*) (PDB ID: 3bl6) and Gram-negative bacteria (*E. coli*) (PDB ID: 1c14) were downloaded from (http://www.rcsb.org./pdb). The proteins were prepared for docking using Discovery Studio, which added hydrogen and disulfide bonds to PDB and eliminated small molecules and water molecules (greater than 5A radius) from the structure. The energy minimization algorithm of the Molecular Operating Environment (MOE2022 software) was utilized to minimize the energy of the coordination compounds and protein molecules. Then, DAC@TCH, and Amoxicillin (ligands) are prepared for docking. The energy of the ligand molecule was minimized using the energy minimization algorithm of *Molecular Operating Environment (MOE) 2022.02 (Chemical Computing Group, Montreal, Canada*; https://chemcomp.com))^55^*.* The binding of the ligand molecule with the protein molecule was analyzed using the MOE docking program to find the correct conformation (with the rotation of bonds, the structure of the molecule is not rigid)^56^.

### Physicochemical, drug-likeness and in silico toxicity

The drug-likeness and potential toxicity of a molecule are determined by its physicochemical properties. The ADME process, which includes absorption, distribution, metabolism, and excretion, plays a crucial role in defining a chemical’s drug-like characteristics, describing how it enters, circulates through, is processed by, and leaves the body. To predict these attributes, computational methods are employed to assess a compound’s ability to cross cell membranes, engage with transporters and enzymes involved in drug uptake and elimination, and maintain metabolic stability. In our study, we utilized ADME prediction tools such as Swiss ADME to examine these drugs. These platforms enabled us to investigate physicochemical attributes and drug similarity behavior. To evaluate toxicity levels, we employed the Pro-Tox II web tool. This program uses statistical algorithms to compare a substance’s molecular structure against an extensive database of known toxic compounds, estimating the likelihood of causing toxicity or adverse effects in humans or other organisms. It provides data on LD50 values, toxicity classifications, and specific toxicological endpoints including cytotoxicity, ecotoxicity, and nutritional toxicity^57^.

### Sample analysis

Surface water samples were collected from the Bohia intake of Sinbellawien Water Station, tap water was obtained from EL-Mansoura city, and groundwater samples were collected from Belbeis Desert. A sintered glass G4 filter was used to filter each sample. All the chosen samples were then preserved in polyethylene containers for later use after being acidified to pH 2 with concentrated nitric acid. Prior to separation, the organic matter was digested; one liter of the chosen water sample was combined with 0.5–1.0 g of K_2_S_2_O_8_ and heated for 30 min at 95 °C. In a series of transparent stoppered bottles with varying concentrations of metal ions at 25 °C and ideal pH conditions, 0.00 and 5 g.l^−1^, 50 mg of DAC@TCH was added after cooling to room temperature. For 120 min, the stoppered bottles were stirred at 150 rpm on an adjusted shaker before being filtered.

### Statistical analysis

At least, three replications of the experimental tests were carried out, and the mean value and its ± standard deviation (SD) are shown as the results.

## Results and discussion

### Materials’ design

#### Synthesis of dialdehyde cellulose (DAC)

The synthesis of DAC was accomplished by selective oxidation of the two adjacent secondary hydroxyl groups that are present on C_2_ and C_3_ in the glucopyranose units in cellulose chains, yielding the ring-opened product (DAC) containing two aldehyde groups as illustrated in (Fig. 1), with high selectivity and yield. The Aldehyde content (AC (%)) is a measure of the degree of oxidation (Amount of reacted monosaccharide units with periodate)^58^. The AC (%) for DAC was found to be 39.50%.

#### Preparation of DAC@TCH

After oxidation, TCH reacted with DAC at 353 K to form DAC@TCH. The DAC@TCH is a solid yellowish powder. 1 g of DAC@TCH was suspended in 50.0 mL of DDW to determine the substance’s solubility in water. The amount of DAC@TCH that resulted from stirring the suspension for about 3.0 h was filtered out and dried. The synthetic steps of DAC@TCH are shown in (Fig. 1).

### Characterization of the polymeric samples

#### Elemental analysis

Table 2 displays the outcomes of the elemental analysis conducted on native cellulose, DAC, and DAC@TCH. The appearance of nitrogen (constituting 21.75% of the total) and sulfur (11.26% of the total) in DAC@TCH validates the successful integration of thiocarbohydrazide moieties into DAC. The analysis suggests that the incorporated TCH units were present at a concentration of approximately 2.4376 mmol/g.

**Table 2:** Elemental analysis of row cellulose, DAC and DAC@TCH.

Fibers	C (%)	H (%)	N (%)	S (%)
Native cellulose	41.55	6.05	–	–
DAC	40.99	6.02	–	–
DAC@TCH	38.71	5.94	21.75	11.26

#### SEM analysis

**Fig. 2:**
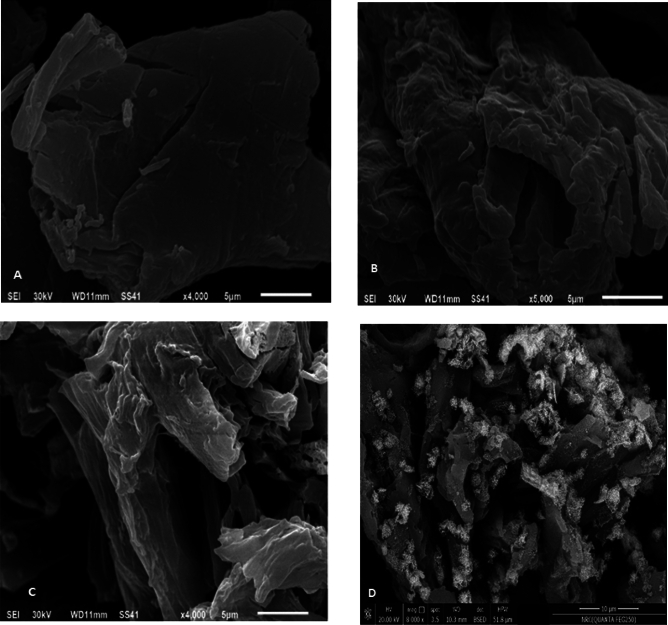
SEM photos of (**a**) cellulose, (**b**) DAC, (**c**) DAC@TCH, and (**d**) DAC@TCH-Cu^2+^ after adsorption of Cu^2+^.

SEM analysis (depicted in Fig. 2 was employed to observe the surface morphology of native cellulose, DAC, and DAC@TCH, as well as DAC@TCH-Cu^2+^. When cellulose is oxidized by KIO_4_ (Fig. 2b), the surface of the DAC has more tights and powder aggregates than that of the native cellulose (Fig. 2a)^43^. In terms of DAC (oxidized cellulose), the DAC@TCH surface morphology has not changed significantly, indicating that the ordered structure of cellulose powder was kept after the addition of thiocarbohydrazide moieties (Fig. 2c). Cu^2+^ ions adsorbed on DAC@TCH (as an example) increase surface roughness and brightness than the other samples because the Cu^2+^ has better electric conductivity than the DAC@TCH and exhibits bright copper (Cu) ions with a low number which have a uniform and homogenous distribution on the surface of DAC@TCH adsorbent (Fig. 2d)^59^.

#### Transmission electron microscopy (TEM)

**Fig. 3:**
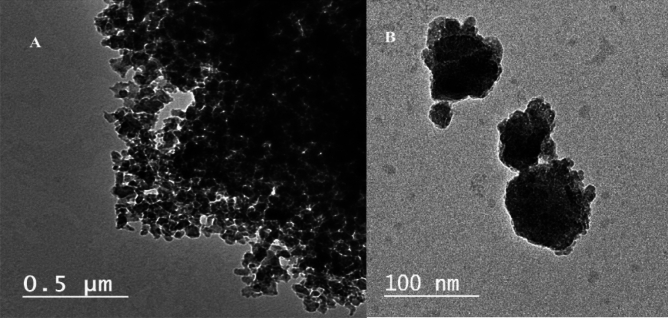
TEM of DAC@TCH at different scales (**a**) 0.5 µm and (**b**) 100 nm.

The TEM of DAC@TCH in different scales, Fig. 3a,b, showed that the DAC@TCH samples were spherical particles of average size less than 100 nm.

#### Infrared spectra

**Fig. 4:**
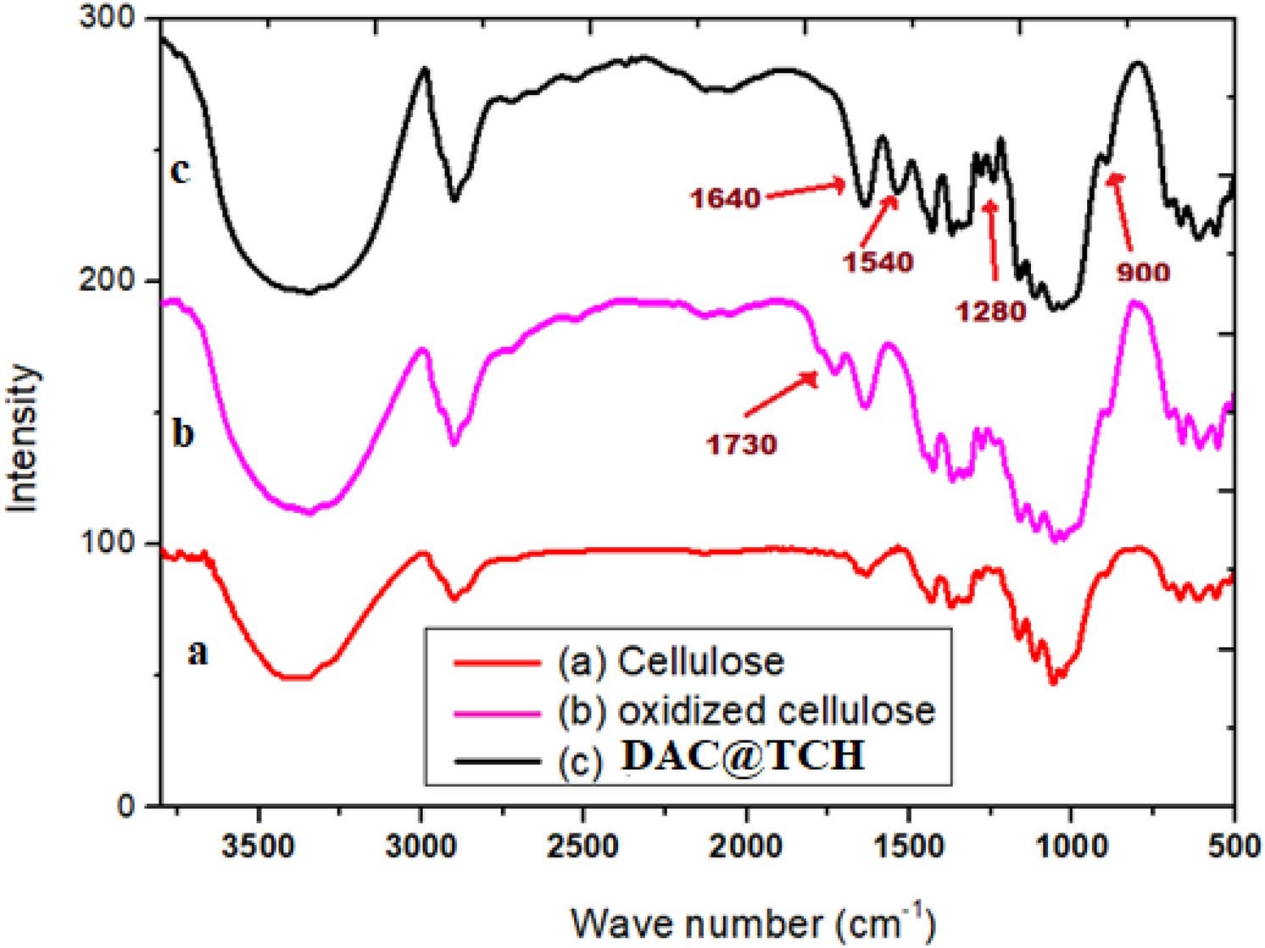
FTIR spectra of (**a**) native cellulose, (**b**) DAC and (**c**) DAC@TCH.

The subsequent steps for synthesis of DAC@TCH were investigated by using FT-IR spectra and the findings are presented in (Fig. 4).

**For native cellulose**, the infra-red spectrum (Fig. 4a) showed the appearance of new peaks at approximately 1070–1150 cm^−1^ that may be returned to C–O stretching vibrations, while at 1250–1420 cm^−1^ and 3200–3500 cm^−1^ may be attributed to OH bending and stretching vibrations, respectively.

**For DAC,** the infra-red spectrum (Fig. 4b) showed an apparent peak of around 1730 cm^−1^, associated with the newly developed aldehyde stretching vibrations group^60^.

**The infra-red spectrum of DAC@TCH** (Fig. 4c), exhibits new relative peaks at 1280 and 900 cm^−1^ that may be returned to the S=C group of the TCH ligand. In addition, peaks are detected at ~ 1640 cm^−1^ and 1540 cm^−1^ which may be attributed to stretching vibrations of the N=C group and bending vibrations for the N–H bond of the Schiff base that was established between the DAC (C=O) groups and the TCH (-NH_2_) groups^61^.

The characterization findings, especially SEM and FTIR findings confirm the formation of DAC@TCH through two basic steps. At first, the native cellulose was converted to DAC (oxidized cellulose form); then followed by modification with the Thiocarbohydrazide chelating ligand (TCH). The modification occurred through the reaction of -NH_2_ active groups of the TCH with C=O of DAC.

#### Thermogravimetric analysis (TGA and DTA)

**Fig. 5:**
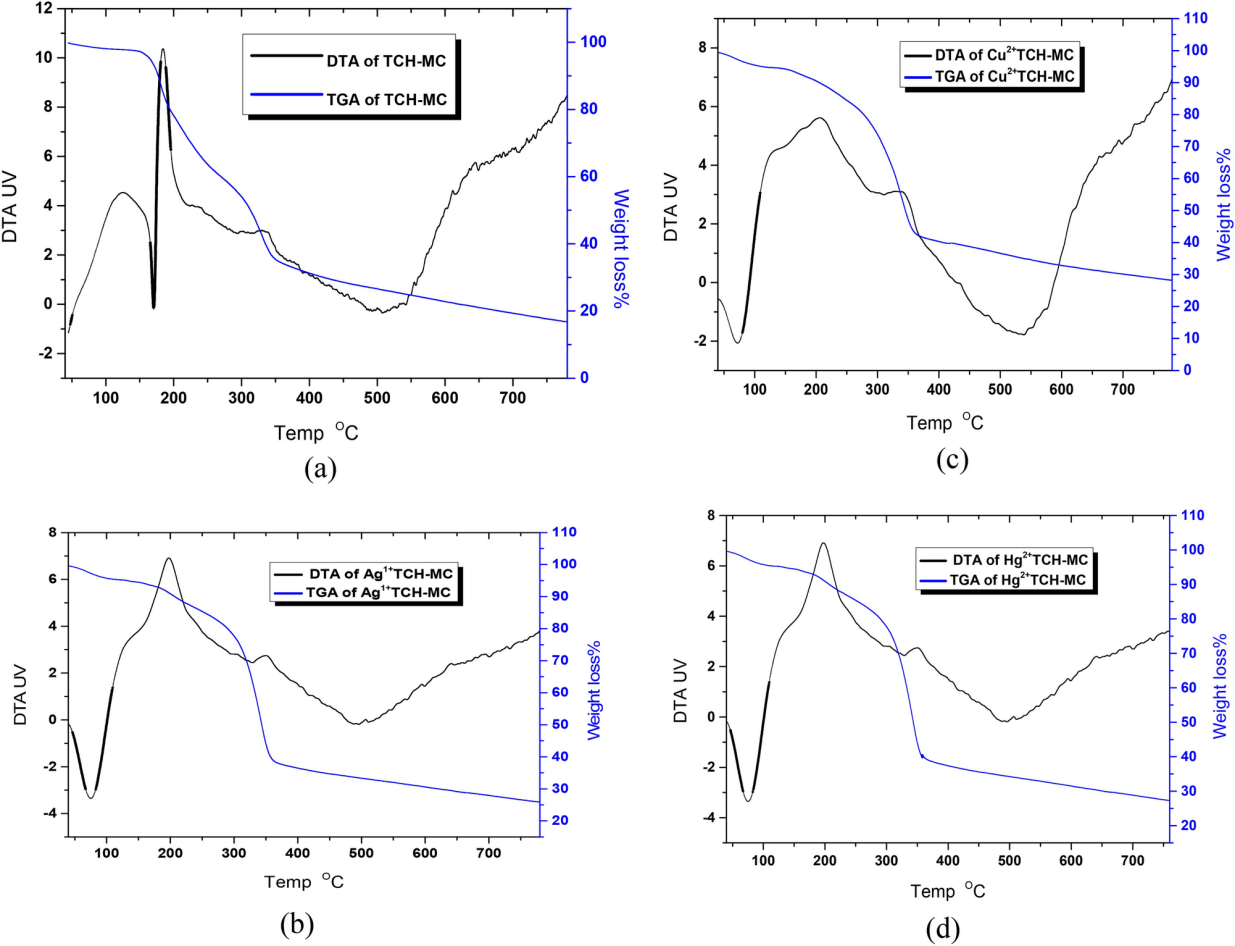
TGA and DTA analysis of (**a**) DAC@TCH (**b**) DAC@TCH-Ag^+^, (**c**) DAC@TCH-Cu^2+^, and (**d**) DAC@TCH-Hg^2+^.

The TGA and DTA analyses were executed on modified cellulose samples at the temperature range from 30 to 800 °C to estimate the thermal stability of cellulosic s. The degradation processes that each compound goes through were recorded using thermograms. Two stages of native cellulose decomposition are revealed by TGA. On the other hand, four decomposition steps are shown on the thermogram of DAC@TCH, confirming the modification step of DAC. Under the same conditions, the thermal degradation of DAC@TCH complexes with the metal ions (Cu^2+^, Ag^+^, and Hg^2+^) was investigated. The total residue was found to be 16.1, 27.79, 25.03, and 26.2%, for DAC@TCH-Ag^+^, DAC@TCH-Hg^2+^, and DAC@TCH-Cu^2+^, respectively, as shown in (Fig. 5a–d). The increase in the total metal-chelate residue value when compared to the DAC@TCH value suggests that these metal ions (Cu^2+^, Hg^2+^, and Ag^+^) undergo relatively more stabilization of their metal chelates/metal oxides formation during their complexation with DAC@TCH rather than catalytic degradation^35^*.*

### Biological activity studies of DAC@TCH

The antibacterial activity of DAC@TCH was studied against *E. coli and S. aureus* bacteria by comparing its inhibition zones with the inhibition zones of Amoxicillin. As presented in (Table 3 & Fig. S1), DAC@TCH showed stronger activity than Amoxicillin antibiotic against *E. coli* bacteria.

**Table 3:** Biological activity studies of DAC@TCH.

Compound	Type of solvent	Gram-negative		Gram-positive	
		*E. coli*		*S. aureus*	
		IZD (mm)	A.I (%)	IZD (mm)	A.I (%)
DAC@TCH	Water	28	560	17	77.3
Amoxicillin		5	100	22	100
DAC@TCH	DMSO	27	540	17	77.3
Amoxicillin		5	100	22	100

### Computational studies

#### Optimized geometry

**Fig. 6:**
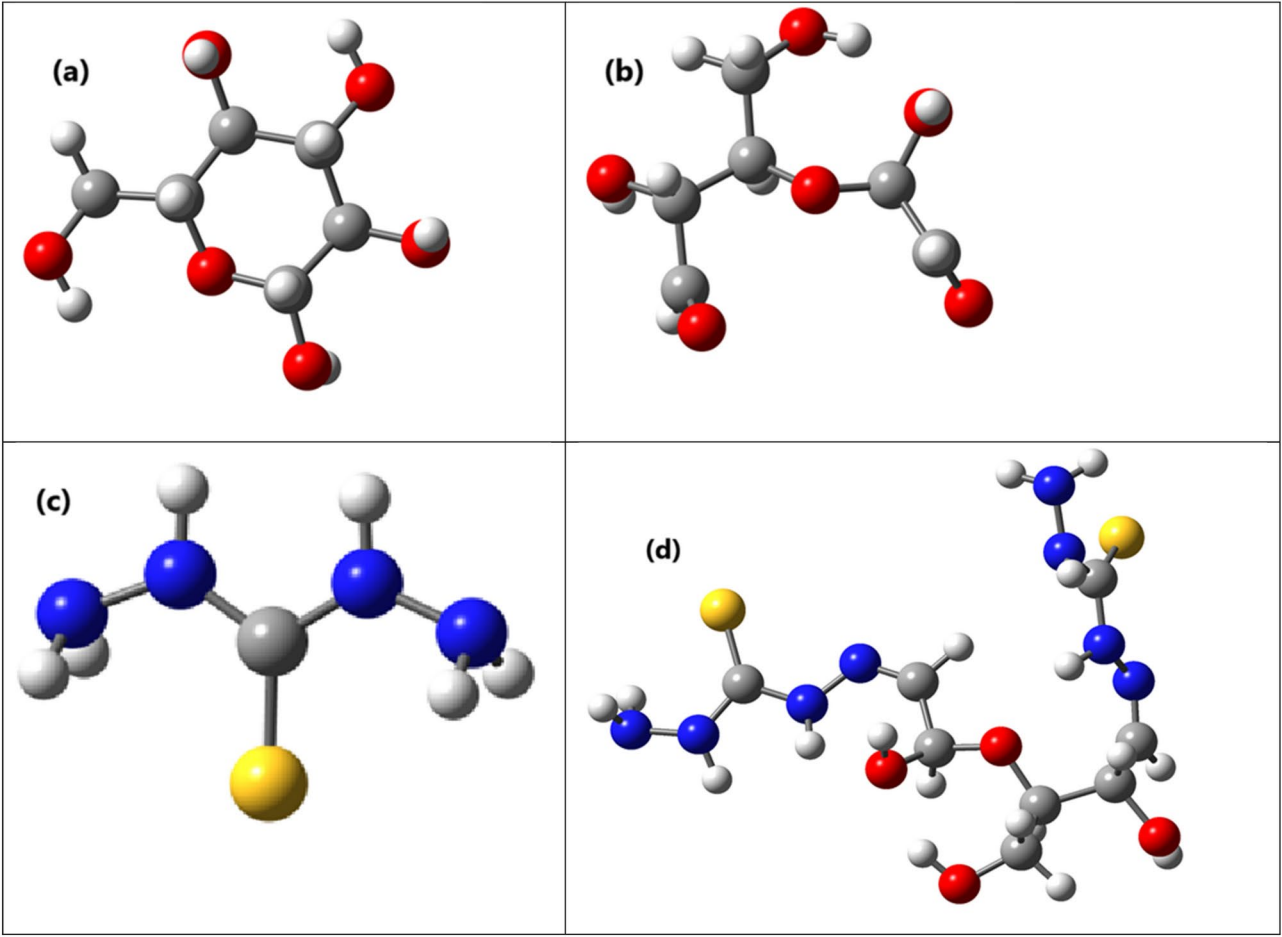
The optimized structures for the (**a**) Cellulose, (**b**) Dialdehyde cellulose (DAC), (**c**) Thiocarbohydrazide (TCH), and (**d**) Modified cellulose (DAC@TCH) based on the DFT/ B3LYP/6–311 + g(d,p) methodology.

The optimized geometry of the cellulose unit, dialdehyd cellulose (DAC), thiocarbohydrazide (TCH) and modified cellulose (DAC@TCH) are shown in (Fig. 6).

#### Global Reactivity Descriptors

**Fig. 7:**
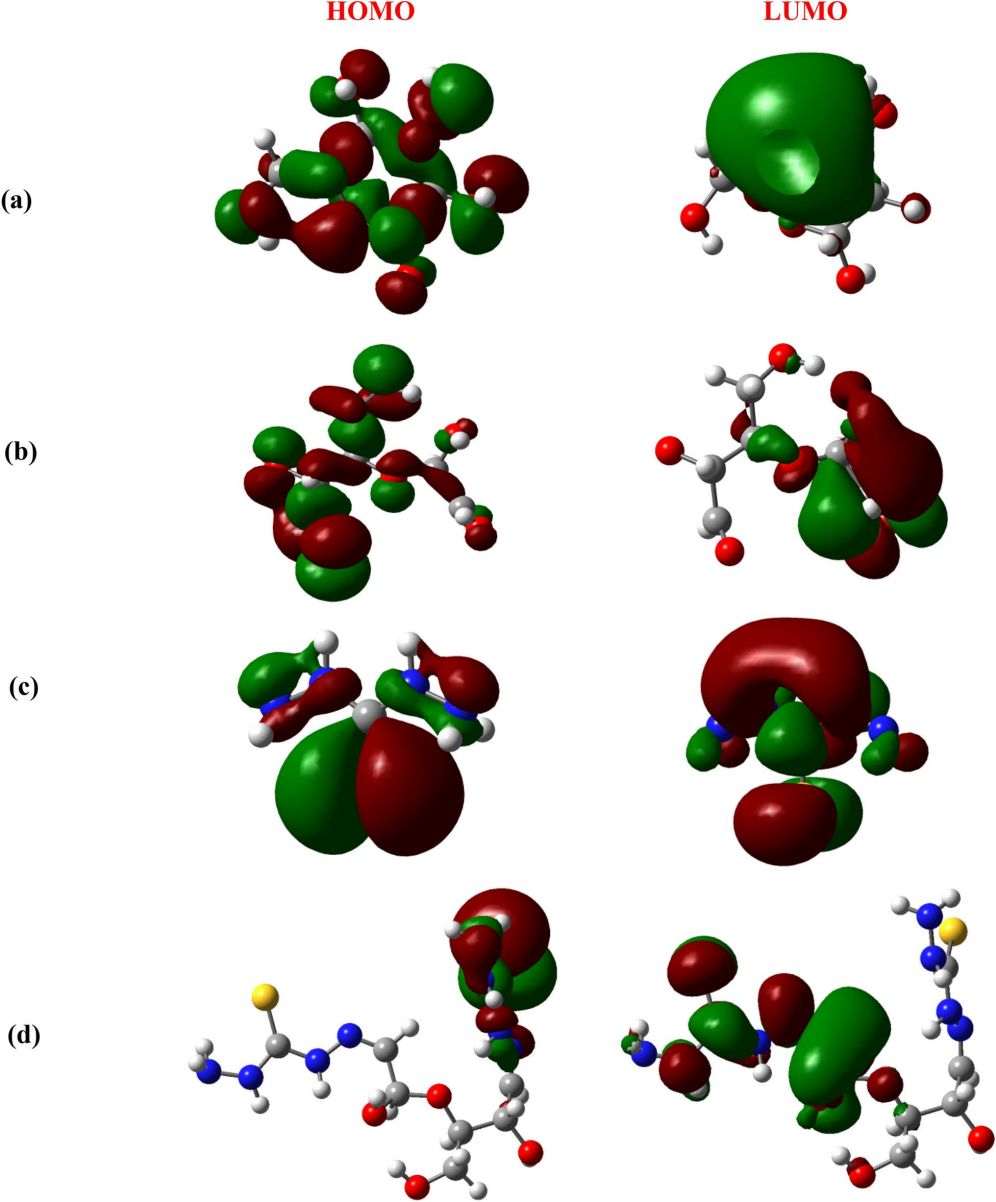
HOMO and LUMO structures for the (**a**) Cellulose, (**b**) Dialdehyde cellulose (DAC), (**c**) Thiocarbohydrazide (TCH) and (**d**) Modified cellulose (DAC@TCH) based on the DFT/ B3LYP/6–311 + g(d,p) methodology.

The global reactivity parameters^62–64^ of a compound can be predicted from the HOMO–LUMO gap. The HOMO is electron donor and LUMO the electron acceptor sites are shown in Fig. 7 for the cellulose unit, dialdehyde cellulose (DAC), thiocarbohydrazide (TCH) and the modified cellulose (DAC@TCH). From Table 4, the higher reactivity of the modified cellulose (DAC@TCH) over the cellulose is explained in the light of energy gap, ΔE_gap_, which measures the reactivity; as the energy gap decreases the reactivity increases. Also, the reactivity of the modified cellulose (DAC@TCH) over the dialdehyde cellulose (DAC) and the thiocarbohydrazide (TCH) is cleared from the results of their ΔE_gap_ and the amount of electronic charge transferring. Table 4 illustrates that, ΔE_gap_ for the modified cellulose (DAC@TCH) was found to be more reactive than of the cellulose and the dialdehyde cellulose (DAC) also was found to be more reactive than the thiocarbohydrazide (TCH). Hard molecules ($\eta )$ have a large energy gap, and soft molecules ($\sigma$) have a small energy gap^23,24^. A soft molecule is more reactive than a hard molecule because a soft molecule has a lower ΔE_(LUMO–HOMO)_^65^. As seen from Table 4 the modified cellulose (DAC@TCH) is softer than cellulose or dialdehyde cellulose (DAC) and thiocarbohydrazide (TCH). This confirms that the modified cellulose (DAC@TCH) is more reactive than the cellulose or the dialdehyde cellulose (DAC) and the modified cellulose (DAC@TCH) is more reactive than thiocarbohydrazide (TCH). The χ is a measure of power of atom(s) to attract the electrons from the other molecules^66^. A high value of electronegativity (χ) for the methylene blue suggests strong ability to attract electrons from the modified cellulose (DAC@TCH), which leads to greater interaction to form the compound.

**Table 4:** The global reactivity parameters determined using the DFT/ B3LYP/6–311 + g(d,p) methodology of calculations for the cellulose, dialdehyde cellulose (DAC), thiocarbohydrazide (TCH) and the modified cellulose (DAC@TCH).

Property	Cellulose	DAC	TCH	DAC@TCH
E_HOMO_ [eV]	−0.27824	−0.27377	−0.22583	−0.22372
E_LUMO_ [eV]	−0.02454	−0.06974	−0.01905	−0.07252
E_gap_ [eV]	0.2537	0.20403	0.20678	0.1512
[eV]	0.12685	0.102015	0.10339	0.0756
[eV]^−1^	7.883327	9.80248	9.672115	13.22751
I_p_ [eV]	0.27824	0.27377	0.22583	0.22372
E_A_ [eV]	0.02454	0.06974	0.01905	0.07252
[eV]	0.15139	0.171755	0.12244	0.14812
µ [eV]	−0.15139	−0.171755	−0.12244	−0.14812
ω [eV]	0.0903387	0.1445855	0.0725	0.1451027
The dipole moment [Debye]	1.9853	1.5142	2.6849	5.8799

The ionization potential; I_P_ and the electron affinity; E_A_, can be expressed as negative values of E_HOMO_ and E_LUMO_, respectively. Ionization energy is a descriptor expressing the chemical reactivity of atoms and molecules. Higher values of ionization energy indicate higher stability and chemical inertness, and vice versa smaller ionization energy indicates higher reactivity of the atoms and molecules^67^. Table 1 shows the values of the ionization energy of the investigated molecules. The low ionization energy of the modified cellulose (DAC@TCH) indicates its higher reactivity than cellulose or the dialdehyde cellulose (DAC) or thiocarbohydrazide (TCH). According to the definition electrophilicity index (ω) it measures the tendency of chemical species to acquire electrons. The results of electrophilicity seen in Table 4 are in decreasing order; the modified cellulose (DAC@TCH) > the dialdehyde cellulose (DAC) > the thiocarbohydrazide (TCH) > the cellulose. Finally, the dipole moment (μ) is a factor that can also provide information about interaction between molecules. The value (μ) of the modified cellulose (DAC@TCH) is higher than (μ) of the dialdehyde cellulose (DAC) or the thiocarbohydrazide (TCH) or the cellulose itself, this suggests the stronger interactions between the the dialdehyde cellulose (DAC) and the thiocarbohydrazide (TCH) to form the compound. The (E_HOMO_), (E_LUMO_), energy gap ΔE_(LUMO–HOMO)_, ionization potential (IP), electron affinity (EA), hardness ($\eta )$, softness ($\sigma$), electronegativity ($\chi$), chemical potential (μ), The Global electrophilicity index (ω) and dipole moment of the Cellulose, the dialdehyde cellulose (DAC), the thiocarbohydrazide (TCH) and the modified cellulose (DAC@TCH) are listed in (Table 4).

#### Infrared analysis

**Fig. 8:**
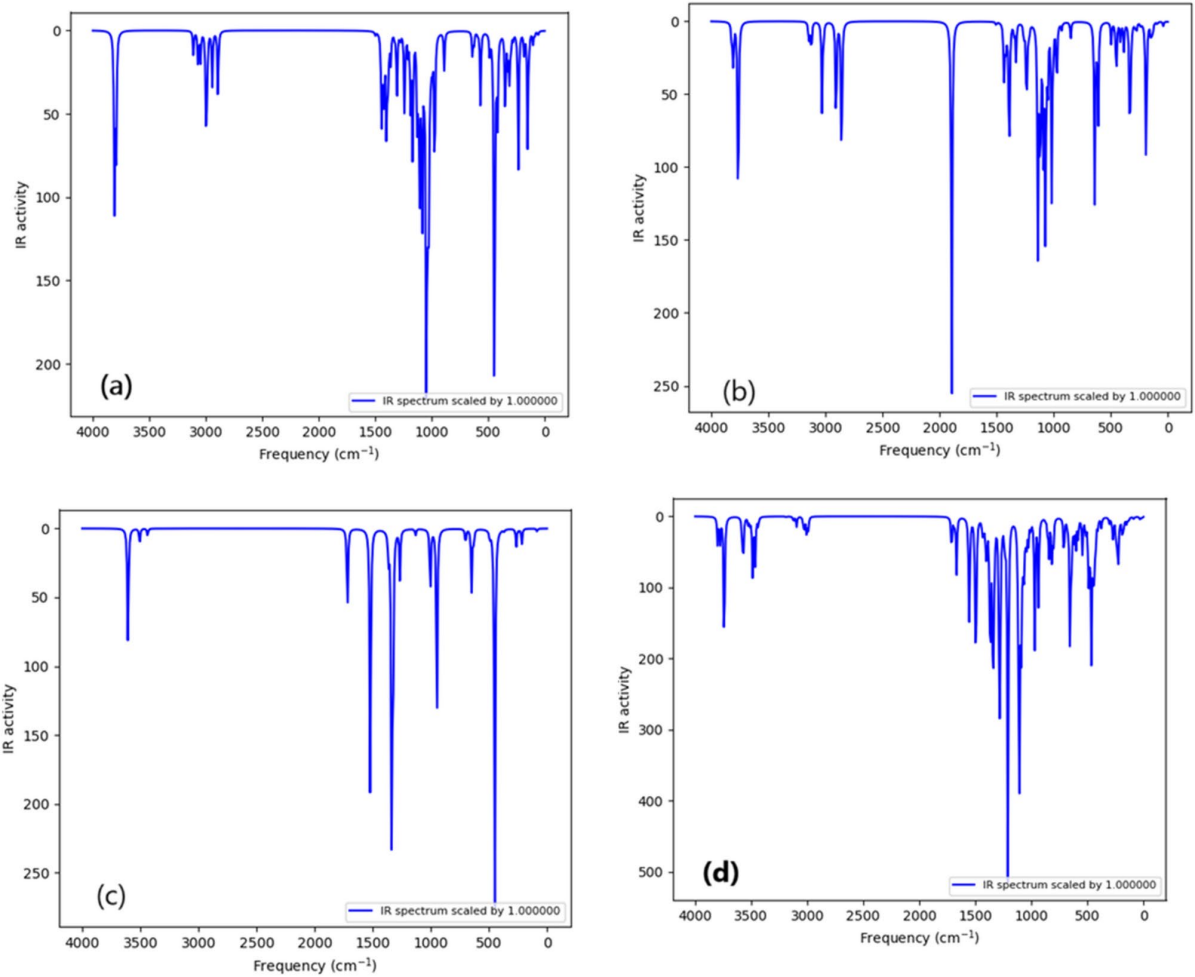
The calculated IR spectrum for the (**a**) Cellulose, (**b**) Dialdehyde cellulose (DAC), (**c**) Thiocarbohydrazide (TCH) and (**d**) Modified cellulose (DAC@TCH) based on the DFT/ B3LYP/6–311 + g(d,p) methodology.

Infrared analyses are supported by theoretical calculations that allow a trustworthy interpretation of experimental spectra of gaseous molecules. B3LYP/6–311 + g(d,p) calculations are the most prominent method used to model IR spectra. The comparison is made between IR spectra calculated for gas phase molecules and those measured in the solid state. Furthermore, Asadi et al. reported a comparison between gas phase DFT and condensed phase experimental data and obtain quite good agreement^68^. The IR spectrum of the Cellulose, the dialdehyde cellulose (DAC), the thiocarbohydrazide (TCH) and the modified cellulose (DAC@TCH) are shown in (Fig. 8).

#### Electrostatic static potential (ESP)

**Fig. 9:**
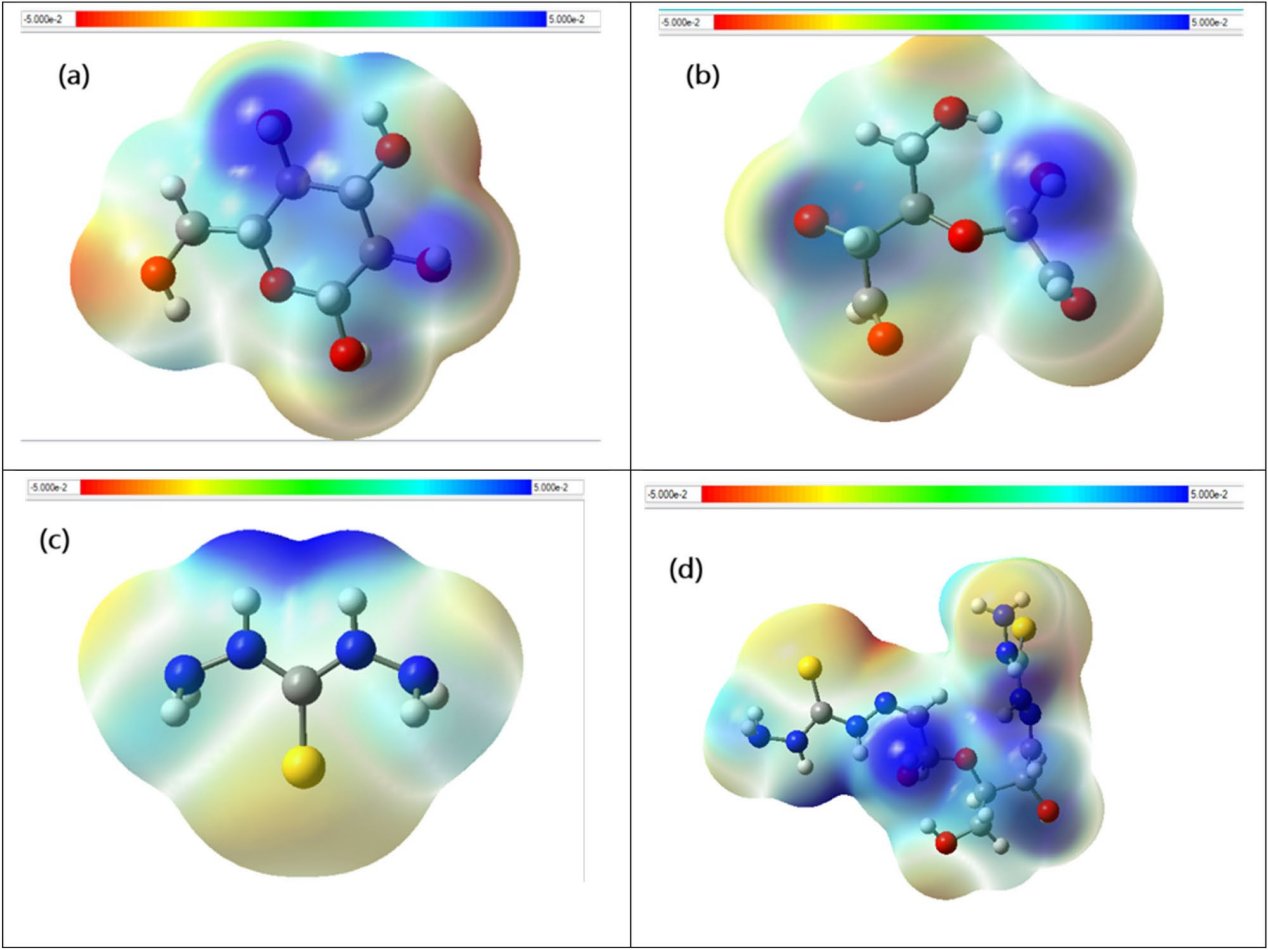
Electrostatic potential (ESP) surface maps visualization for the (**a**) Cellulose, (**b**) Dialdehyde cellulose (DAC), (**c**) Thiocarbohydrazide (TCH) and (**d**) Modified cellulose (DAC@TCH) based on the DFT/ B3LYP/6–311 + g(d,p) methodology. Blue, green and red correspond to ESP varying from min to max level, the blue and red spheres correspond to ESP surface minima and maxima, respectively. ESP ranges are included in the legend at each figure panel.

An electrostatic potential (ESP) surface or map of a molecule shows the partial distribution of change along the molecule’s surface. Electrostatic potential maps, also known as electrostatic potential energy maps, or molecular electrical potential surfaces, illustrate the charge distributions of molecules three dimensionally. These maps allow us to visualize variably charged regions of a molecule. Knowledge of the charge distributions is very useful to help determine molecular polarity and can be used to determine how molecules interact with one another. To make the electrostatic potential energy data easy to interpret, a color spectrum, with red as the lowest electrostatic potential energy value and blue as the highest, is employed to convey the varying intensities of the electrostatic potential energy values. The calculated ESP maps of cellulose, dialdehyde cellulose (DAC), the thiocarbohydrazide (TCH) and the modified cellulose (DAC@TCH) are shown in (Fig. 9).

### Molecular docking studies

**Fig. 10:**
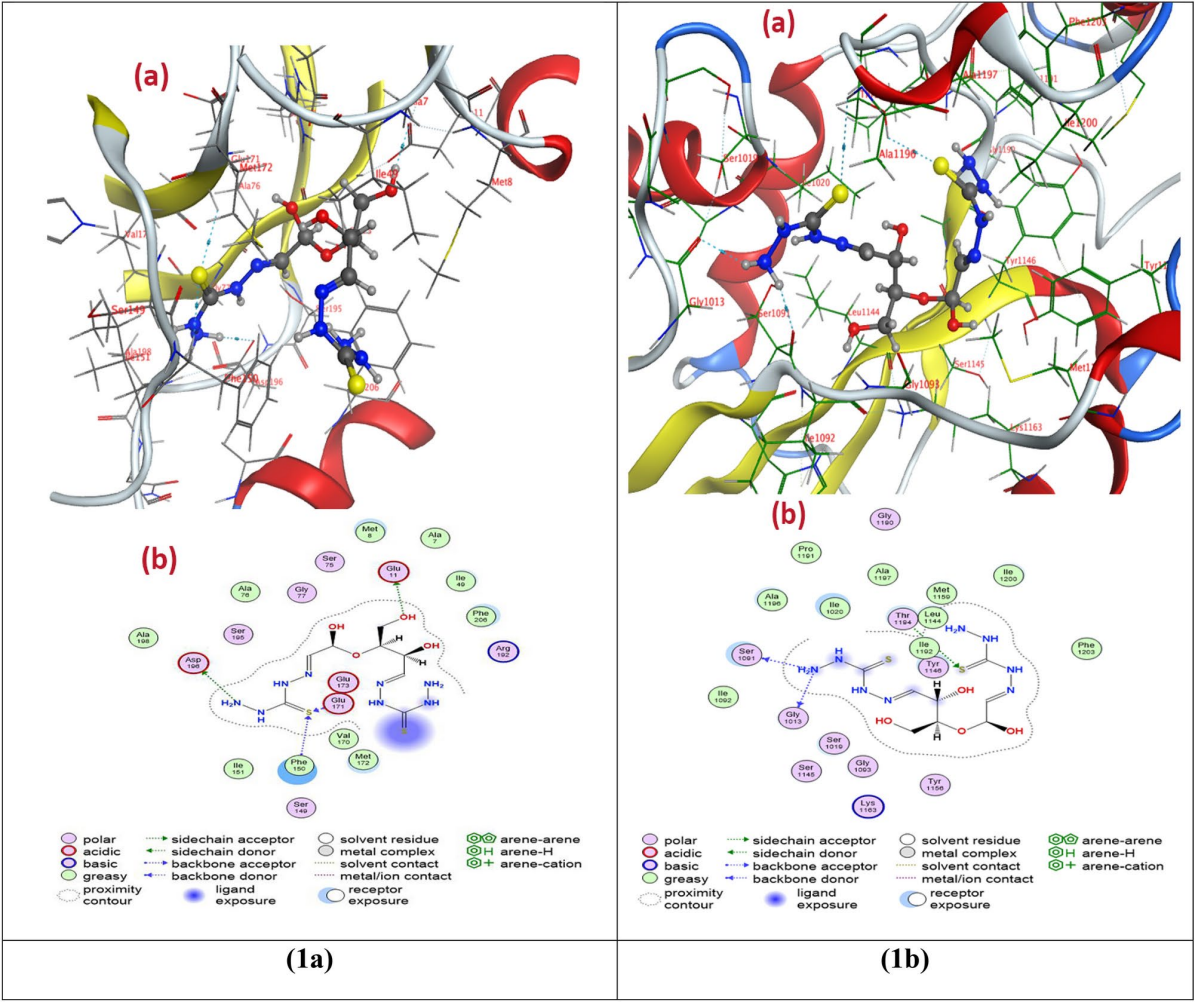

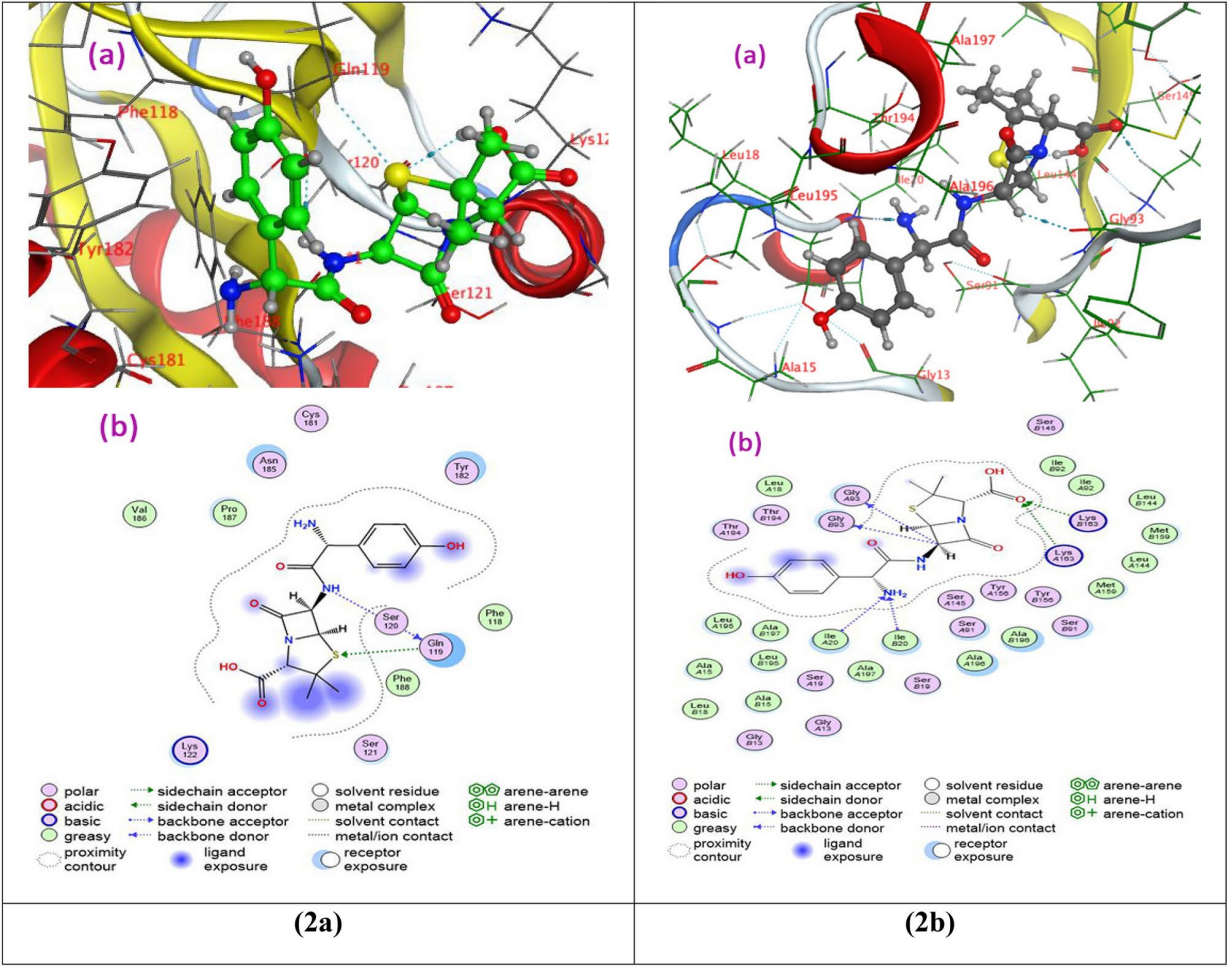
3D (**a**) and 2D (**b**) molecular interaction of (**1a**) DAC@TCH to *S. aureus* & (**1b**) DAC@TCH to *E. coli*, (**2a**) Amoxicillin to *S. aureus* & (**2b**) Amoxicillin to *E. coli*.

Molecular docking studies were performed with the structure of two types of bacteria such as Gram-positive bacteria (*Staphylococcus aureus*) (PDB ID: 3bl6) and Gram-negative bacteria (*E. coli)* (PDB ID: 1c14) to evaluate the preferred binding site of DAC@TCH towards these targets (Figs. 10(1a,b)) and for Amoxicillin (Fig. 10(2a,b)) for comparison, as in vitro studies. From the docking data analysis (Table 5), the binding energies (best docking scores) in kcal/mol for DAC@TCH are (**−**7.4237) and (−7.1325) for *Staphylococcus aureus and, E. coli* respectively meanwhile the binding energies (best docking scores) in kcal/mol for Amoxicillin are (−5.8090) and (−6.7442) for *Staphylococcus aureus and, E. coli* respectively. That means DAC@TCH has a high ability to inhibit the growth of this type of bacteria, which agrees with experimental data shown (Fig. S1) & Table 3 than Amoxicillin**.** Here, the inhibition constant (K*i*) was obtained from the binding energy (ΔG) using the formula: K*i* = exp(ΔG/RT), where R is the universal gas constant (1.985 × 10^−3^ kcal mol^−1^ K^−1^) and T is the temperature (298.15 K). The lower the Ki, the stronger the ligand binds to the target, implying that the ligand is a more potent inhibitor^64,69,70^. So the **lower the binding energy**, the lower the Ki of DAC@TCH than Amoxicillin indicating stronger binding of DAC@TCH to target bacteria than Amoxicillin. Meanwhile Table 6 illustrates the molecular docking interactions predicted for inhibitor binding with *Staphylococcus aureus* and, *E. coli* for DAC@TCH and Amoxicillin.

**Table 5:** Molecular docking best docking scores (S) & rmsd_refine & inhibition constant (K*i*) to two types of bacteria.

Compound	*S. aureus*			*E. coli*		
	best docking scores (S) kcal/mol	rmsd_refine	the inhibition constant (Ki) µM	best docking scores (S) kcal/mol	rmsd_refine	the inhibition constant (Ki) µM
DAC@TCH	−7.4237	1.5308	3.617021524	−7.1325	1.4835	5.912943184
Amoxicillin	−5.8090	1.7968	55.20048571	−6.7442	1.9977	11.38758478

**Table 6:** Molecular docking interactions predicted for inhibitor binding to two types of bacteria.

Compound	* S. aureus*		*E. coli*	
	Hydrogen bonds		Hydrogen bonds	
	Donor acceptor	Type	Donor acceptor	Type
DAC@TCH	NH_2_ → ASP196	Side chain doner	NH_2_ → Ser1091	Backbone donor
	Glu171 → S	Backbone acceptor	NH_2_ → Gly 1013	Backbone donor
	Phe 150 → S	Backbone acceptor	Thr1194 → S	Side chain acceptor
	OH → Glu11	Side chain doner		
Amoxicillin	NH → Gln119	Backbone doner	C → GlyA93	Backbone doner
	Gln119 → S	Side chain acceptor	C → GlyB93	Backbone doner
			Lie A20 → NH_2_	Backbone acceptor
			Lie B20 → NH_2_	Backbone acceptor
			LysA163 → O	Side chain acceptor
			LysB163 → O	Side chain acceptor

Computational drug-likeness evaluations play a crucial role in accelerating drug discovery by efficiently identifying and prioritizing promising candidates. These digital tools streamline processes, reduce costs and experimental burden, and facilitate the early identification of compounds with desirable pharmacokinetic properties and target interactions. Researchers can leverage these tools to rapidly narrow down vast compound libraries to a handful of potential candidates, thereby expediting the drug development process.

The Lipinski rule of five delineates specific physicochemical properties essential for human oral bioavailability: no more than 5 hydrogen bond donors, no more than 10 hydrogen bond acceptors, a maximum of 10 nitrogen or oxygen atoms, a molecular mass below 500 Daltons, and a calculated partition coefficient (MLOGP) not exceeding 4.15. Compliance with these criteria indicates the likelihood of a compound exhibiting oral activity. Notably, all phytoconstituents described in this research comply with Lipinski’s rule of five, as shown in (Table 7). This compliance indicates that these compounds possess the necessary attributes for optimal oral bioavailability. Beyond Lipinski’s rule, the compounds were evaluated against additional drug-likeness criteria, including Veber’s and Egan’s guidelines. Veber’s rule emphasizes the number of rotatable bonds and the topological polar surface area (TPSA), suggesting that compounds with 10 or fewer rotatable bonds and a TPSA of 140 Å^2^ or less are likely to demonstrate good oral bioavailability. Egan’s rule, which examines solubility and permeability, is another important metric. All compounds identified in this study adhere to these additional requirements, further supporting their potential as viable drug candidates^56,63,64^.

**Table 7:** Physico-chemical properties and drug-likeness & toxicity-related parameters.

Characteristics	DAC@TCH	Amoxicillin
Physico-chemical properties and drug-likeness		
Molecular weight (g/mol)	354.41	365.40
Num. heavy atoms	22	25
Num. H-bond acceptors	8	6
Num. H-bond donors	9	4
Num. rotatable bonds	12	5
TPSA (A^2^)	258.98	158.26
Log *P*	−2.36	−0.39
Drug-likeness		
Lipinski*	No; 2 violations: NorO > 10, NHorOH > 5	Yes; 0 violation
Egan**	No; 1 violation: TPSA > 131.6	No; 1 violation: TPSA > 131.6
Veber***	No; 2 violations: Rotors > 10, TPSA > 140	No; 1 violation: TPSA > 140
Bioavailability score	0.17	0.55
Bioavailability radars		
Toxicity-related parameters		
Predicted LD50 (mg/kg)	3200	15000
Predicted toxicity class	5	6
Cytotoxicity Probability	Inactive 0.61	Inactive 0.60
Ecotoxicity Probability	Inactive 0.60	Inactive 0.72
Nutritional toxicity Probability	Inactive 0.52	Active 0.51

*TPSA* topological polar surface area, *LogP* octanol–water partition coefficient.

*Lipinski’s rule of five: MW ≤ 500; HBD ≤ 5; HBA ≤ 10; LogP ≤ 5.

**Egan rule: 0 ≤ LogP ≤ 5.88; TPSA ≤ 131 A2.

***Veber rule: rotatable bonds ≤ 10; TPSA ≤ 140 A.

The drug-likeness of Amoxicillin, and DAC@TCH was evaluated using several criteria, including Lipinski’s, Egan’s, and Veber’s rules. These compounds meet the requirements, suggesting they possess favorable characteristics as potential oral medication candidates. Lipinski’s rule assesses drug-likeness based on molecular weight, lipophilicity, and hydrogen bond donors and acceptors. Egan’s rule focuses on bioavailability, examining solubility and permeability, while Veber’s rule predicts oral bioavailability based on rotatable bonds and TPSA. Amoxicillin have a bioavailability score of 0.55, indicating moderate bioavailability, whereas DAC@TCH has a score of 0.17, suggesting very low bioavailability. These scores, along with bioavailability radar maps, indicate that these compounds have a balanced profile across various crucial factors, including lipophilicity, polarity, insolubility, size, flexibility, and unsaturation. Table 7 provides a comprehensive comparative analysis of the physicochemical properties, drug-likeness, and bioavailability of Amoxicillin, and DAC@TCH. The physicochemical characteristics of Amoxicillin, and DAC@TCH include molecular weights of 365.40, and 354.41 g/mol, and TPSA values of 158.26, and 258.98 Å^2^, respectively. Additional parameters are presented in (Table 7).

According to the toxicity-related parameters, the expected LD50 values for DAC@TCH, and amoxicillin are 3200, and 15000 mg/kg, respectively. In the same order, compounds with predicted toxicity classes of 5, and 6 indicate moderate toxicity. Table 7 illustrates the comparatively low probability values for the different toxic effects of DAC@TCH, including cytotoxicity, ecotoxicity, and nutritional toxicity.

### Adsorption studies

#### Point of zero charge (pH_PZC_)

**Fig. 11:**
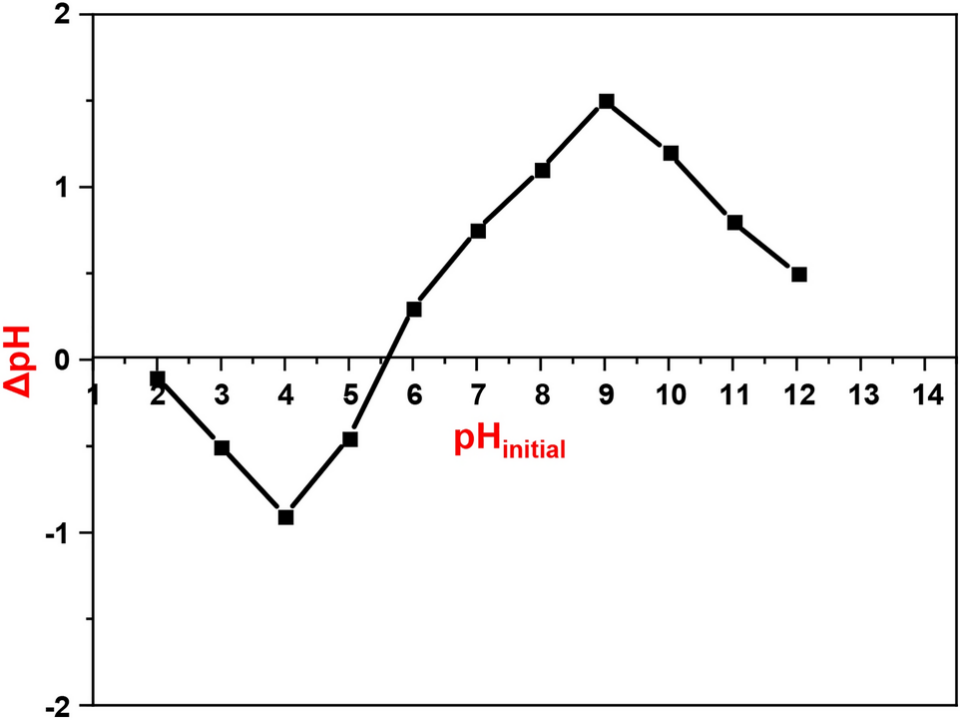
pH_PZC_ for DAC@TCH.

The point of zero charge (pH_PZC_) is a crucial variable for characterizing the sorbate and demonstrating its affinity for the surface of the sorbent. The reported study describes an investigation into the pH_PZC_ of DAC@TCH. The pH was raised from 2 to 12, and the pH_PZC_ of DAC@TCH was calculated. The pH_PZC_ of DAC@TCH is 5.70, (Fig. 11). This value indicates that the surface of the DAC@TCH is neutral at a pH value equal to 5.7, while it is positively charged at pH values lower than 5.7 and is negatively charged at pH values above 5.7.

#### Effect of pH

The adsorption performance of DAC@TCH on metal ions at different pH is shown in (Fig. S2). As it can be noticed with the other constant conditions, as the pH of the solution increases, the adsorption capacities of DAC@TCH for the three metal ions all present a trend of first increasing (pH ≤ 6), and then decreasing (pH > 6). When the pH of the solution was increased from 1 to 6, the adsorption capacities of metal ions onto DAC@TCH increased from 25.2 to 72 mg/g, from 77.4 to 98.5 mg/g, and from 67.4 to 99.6 mg/g for Cu^2+^, Ag^+^, and Hg^2+^, respectively.

The surface sites of DAC@TCH are protonated at a pH lower than pH_PZC_ (5.7) and deprotonated at a pH higher than pH_PZC_ (5.7) value. It was observed that at low pH, the DAC@TCH adsorption efficiency (q_e_) is low and this may be returned to metal ions competing with H + ions for surface sites at this pH. With the increase in the pH, the competition between H^+^ and metal ions for the adsorption sites decreased which leads to high metal ions adsorption. At pH values greater than 6.0, the adsorption efficiencies (q_e_) of metal ions became lower which may be returned to the formation of metal hydroxides within the solution^71^.

#### Effect of DAC@TCH dose

In Fig. S3, the impact of DAC@TCH quantity on the adsorption of Cu^2+^, Hg^2+^, and Ag^+^ is depicted. It’s evident that as the amount of DAC@TCH increases, the removal rates of metal ions initially rise sharply before stabilizing. This phenomenon occurs because the higher quantity of adsorbent offers more available adsorption sites, leading to a reduction in the concentration of residual metal ions and consequently enhancing adsorption efficiency. However, as the amount of DAC@TCH continues to increase, metal ions are essentially adsorbed completely, requiring deeper diffusion to occupy any remaining adsorption sites. Thus, after the further increase of its amount, removal rates all remained unchanged insignificantly. The maximum adsorption capacities of the studied three metal ions were obtained using 0.05 g of DAC@TCH.

#### Effect of the initial metal ion’s concentration and adsorption isotherms

Figure S4 illustrates the impact of the initial concentration (C_0_) of different metal ions on the adsorption performance of DAC@TCH. The adsorption curves for all metal ions exhibit similar trends. As the initial concentration of metal ions in the solution rises, the adsorption capacities of DAC@TCH for various metal ions generally increase. However, this upward trend slows down after reaching a certain concentration. A higher concentration of metal ions translates to a greater absolute content of metal ions per unit volume, increasing the likelihood of contact with DAC@TCH and resulting in a rapid increase in adsorption. Yet, as the initial concentration continues to increase, the adsorption efficiency of DAC@TCH for metal ions saturates, leading to a plateau in the growth rate of the adsorption curve.

Adsorption isotherm models offer a means to scrutinize the mechanism, capacity, and efficacy of adsorbents. Research suggests that the Langmuir and Freundlich models stand out as the optimal fitting models for describing the adsorption of metal ions. Equations (7) and (8).

##### Freundlich

7 $${\text{lnq}}_{{\text{e}}} = {\text{ lnK}}_{{\text{F}}} + 1/{\text{n }}\left( {{\text{lnC}}_{{\text{e}}} } \right)$$

where C_e_ and q_e_ represent the equilibrium concentrations of the adsorbate cation in the liquid phase and the adsorbate in the solid phase, respectively, and K_f_ and n represent the Freundlich coefficients.

##### Langmuir

8 $${\text{C}}_{{\text{e}}} /{\text{q}}_{{\text{e}}} = \left( { \left( {1/\left( {{\text{K}}_{{\text{l}}} {\text{q}}_{m} } \right)} \right) + \left( {{\text{C}}_{{\text{e}}} /{\text{q}}_{m} } \right) } \right)$$

where K_l_ and q_m_ are, respectively, the equilibrium constants for the adsorbate-adsorbent equilibrium and the monolayer capacity represented by Langmuir coefficients.

By plotting (i) ln q_e_ vs. ln C_e_ and (ii) C_e_/q_e_ vs. C_e_, respectively, the linear Freundlich and Langmuir plots are created, from which the adsorption coefficients(R^2^) can be calculated^38,72^.

The obtained experimental data were applied to both the Freundlich and Langmuir isotherm models, and all the parameters were displayed in (Fig. S5 and Table 8).

**Table 8:** Parameters of adsorption isotherm models.

System	Langmuir isotherm constants			
	K_L_(L/g)	q_m_(mg/g)	R^2^	R_L_
DAC@TCH–Cu^2+^	2.25	71.9	0.9991	9.685*10–3
DAC@TCH–Ag^+^	2.14	196	0.9993	4.834*10–3
DAC@TCH–Hg^2+^	5.84	190	0.9992	4.9*10–3

System	Freundlich isotherm constants			
	K_F_	n	R^2^	
DAC@TCH–Cu^2+^	43.29	9.12	0.712	
DAC@TCH–Ag^+^	91.84	4.73	0.318	
DAC@TCH–Hg^2+^	112.5	6.53	0.387	

Table 8 demonstrates that the correlation coefficients (R^2^) of the Langmuir models for Cu^2+^, Ag^+^, and Hg^2+^ were consistently higher than those of the Freundlich models, with R^2^ values exceeding 0.9990. This suggests that the adsorption behavior of DAC@TCH for Cu^2+^, Ag^+^, and Hg^2+^ conforms more closely to the Langmuir isotherm model. Specifically, it indicates that Cu^2+^, Ag^+^, and Hg^2+^ are adsorbed in a monolayer on the DAC@TCH adsorbent surface, with maximum adsorption capacities of 73, 194, and 190 mg/g for Cu^2+^, Ag^+^, and Hg^2+^, respectively. The Langmuir adsorption isotherm model’s characteristics can be summarized using a dimensionless equilibrium parameter, R_L_, where R_L_ = 1/(1 + KLC_0_)^73^, with C_0_ representing the initial metal ion concentration. Utilizing the highest initial metal ion concentration (300 mg/L), RL values for the adsorption of Cu^2+^, Ag^+^, and Hg^2+^ fell within the favorable range of 0–1. This suggests that their adsorption processes were favorable, consistent with the RL results. Given the superior correlations of the Langmuir isotherm adsorption model fitting, it can be inferred that the adsorption processes of DAC@TCH for Cu^2+^, Ag^+^, and Hg^2+^ involved monolayer adsorption.

#### Effect oscillation time and adsorption kinetics

The impact of contact time on the adsorption of Hg^2+^, Cu^2+^, and Ag^+^ onto the DAC@TCH was exhibited in (Fig. S6). Initially, both adsorption capacity and removal percentage increased rapidly, indicating a high availability of active adsorption sites on the DAC@TCH surface, leading to accelerated adsorption rates. However, as time progressed, a saturation point was reached where most of the available adsorption sites became occupied by Hg^2+^, Cu^2+^, and Ag^+^ resulting in a reduction in adsorption effectiveness and speed. Consequently, the removal efficiency and adsorption capacity remained relatively unchanged, indicating equilibrium. Selecting an appropriate adsorption equilibrium time can effectively shorten the adsorption process. In this study, the equilibrium time for Hg^2+^, Cu^2+^, and Ag^+^ adsorption was determined to be 120 min.

The adsorption kinetics experiments were obtained at the optimum conditions of the Ag^+^, Cu^2+^, and Hg^2+^ adsorption onto the DAC@TCH to investigate the adsorption effect at different oscillation times. The pseudo-1st-order Eq. (9) and the pseudo-2nd-order kinetic models Eq. (10) were employed to match the data for a better understanding of the adsorption kinetic process of the Ag^+^, Cu^2+^, and Hg^2+^ions onto DAC@TCH.

9 $$1/{q}_{t(ads)}={K}_{1}/{q}_{e(ads)}t+ 1/{q}_{e(ads)}$$

10 $$t/{q}_{t(ads)}=1/{K}_{2}{{q}_{e}}_{(ads)}^{2}+ (1/{q}_{e(ads)})t$$

where, K_2_ and K_1_ are the rate constants for the pseudo-2^nd^-order and the pseudo-1st-order, respectively. While q_e_ and q_t_ that are expressed in mg/g can be defined as the adsorption capabilities at equilibrium and at time t, respectively.

K and q_e_ parameters were usually estimated together for the two studied models in addition to the correlation coefficient (R^2^) was utilized to test the well-fitted kinetic model for the experimental work. The R^2^ values of non-linear curves are more realistic and accurate to estimate the kinetic parameters. Both Fig. S7 and Table 9 show non-linear regression findings of the pseudo-1st-order and pseudo-2^nd^-order kinetic models. R^2^ for the pseudo-2^nd^-order kinetic model for Hg^2+^, Ag^+^, and Cu^2+^ (R^2^ > 0.9900) were mostly higher than those of the pseudo-1st-order model, reflecting a better fit of the non-linear regression curves for pseudo-2nd-order kinetics and that the adsorption process could be due to chemisorption mechanism^73,74^.

**Table 9:** DAC@TCH adsorption of Cu^2+^, Ag^+^, and Hg^2+^ kinetic parameters.

System	Pseudo-first-order		
	k_1_ (min^−1^)	*q*_*e1ads*_ (mg/g)	R^2^
DAC@TCH–Cu^2+^	71.2	125	0.929
DAC@TCH–Ag^+^	6.39	105.2	0.920
DAC@TCH–Hg^2+^	7.1	107.5	0.856

System	Pseudo-second-order		
	K_F_	n	R^2^
DAC@TCH–Cu^**2+**^	2.64 × 10^–4^	88.5	0.992
DAC@TCH–Ag^+^	2.8 × 10^–3^	102	0.999
DAC@TCH–Hg^2+^	2.8 × 10^–3^	102	0.999

#### Thermodynamic studies

Specific parameters such as standard free energy (ΔG^o^_ads_), enthalpy heat (ΔH^o^_ads_), and adsorption entropy (ΔS^o^_ads_) with DAC@TCH for Hg^2+^, Cu^2+^, and Ag^+^ metal ions adsorption with DAC@TCH were calculated by adsorption of the desired metal ions at temperatures ranging from 293 to 318 K.

The thermodynamic reaction rate constant (Kc) and parameters were calculated as follows:

11 $${K}_{c}= {C}_{ad}/{C}_{e}$$

where Cads denotes the metal ion concentration sorbed on the DAC@TCH at equilibrium (mg/g) and Ce denotes the concentration at equilibrium (mg/L).

12 $${\Delta G}_{ads}^{o}= -RTln{K}_{C}$$

13 $$lnK_{C} = \left( {\Delta S_{ads}^{o} /R} \right) {-} \left( {\Delta H_{ads}^{o} /RT} \right)$$

where (8.314 J/mol K) is the universal gas constant (R). The slope (H^o^_ads_/R) and intercept (S^o^_ads_/R) of the plot of ln Kc vs 1/T were used to determine the values of H^o^_ads_ and S^o^_ads_., as shown in (Fig. S8). From the evaluated thermodynamic parameters that present in Table 10, it was indicated that the (−ve) value of ΔG^o^_ads_ demonstrates that the spontaneous adsorption of metal ions by DAC@TCH at room-temperature. Also, the negative value of ΔH^o^_ads_ means that the process of adsorption is exothermic in nature and some amount of heat is lost upon the adsorption of metal ions. However, as a result of the adsorption of metal ions to DAC@TCH, the negative values of ΔS^o^_ads_ show the system’s higher alignment and lower randomness^53^. Due to the lower interaction between the metal ions and the active groups in DAC@TCH, the adsorption capacity of the metal ions decreased by increasing the temperature.

**Table 10:** Thermodynamic parameters of Hg^2+^, Cu^2+^, and Ag^+^ single metal ions adsorbed onto DAC@TCH.

System	K_c_				ΔG^o^_ads_ (KJ/mol)				ΔH^o^_ads_ (KJ/mol)	ΔS^o^_ads_ (J/mol K)
	293 K	298 K	308 K	318 K	293 K	298 K	308 K	318 K		
DAC@TCH–Cu^2+^	70	49	26.7	13.08	−10.35	−9.638	−8.419	−6.794	−51.249	−139.5
DAC@TCH–Ag^+^	12.8	11.7	9.5	7.4	−6.210	−6.093	−5.764	−5.291	−17.024	−36.69
DAC@TCH–Hg^2+^	142	110	65.6	32	−12.07	−11.65	−10.71	−9.162	−46.731	−117.6

#### Effect of interfering ions

Different ions were used at the metal ions removal by DAC@TCH optimum conditions, to investigate the provided method selectivity. The experimental results in (Table 11, Table 12) explain that there is no obvious effect on the determination of the metal ions tested under the optimum working conditions defined in the procedure. It can be deduced that the provided technique has a high selectivity toward the metal ions separation and determination in different actual water samples.

**Table 11:** Effect Interfering ions on the recovery percentage value and limits of tolerance (Conditions: pH = 6, 500 mg dose, at 298 k, *n* = 3).

Ions	Tolerance limit (mg/L)	Cu^2+^		Ag^+^		Hg^2+^	
		Recovery (%)	SD*	Recovery (%)	SD	Recovery (%)	SD
Na^+^	1000	99.0	0.13	99.3	0.17	98.3	0.23
K^+^	1000	98.1	0.21	98.7	0.07	98.2	0.34
Mg^2+^	500	97.8	0.16	98.4	0.12	98.2	0.1
Ca^2+^	500	97.8	0.09	99.1	0.1	99.0	0.131
Al^3+^	50	97.5	0.18	96.4	0.134	95.7	0.65
PO_4_^3−^	500	99.0	0.1	98.2	0.16	99.0	0.12
Acetate	50	97.0	0.18	98.7	0.13	98.8	0.23
Oxalate	50	98.1	0.11	98.0	0.15	97.2	0.17
Citrate	50	96.8	0.24	95.4	0.11	94.6	0.135
NO_3_^−^	200	99.2	0.08	99.5	0.21	99.9	0.14
Cl^−^	500	97.8	0.14	97.9	0.174	98.7	0.11
HCO_3_^−^	1000	96.5	0.16	98.3	0.24	98.0	0.24
SO_4_^2−^	1000	99.2	0.09	98.0	0.14	99.7	0.13
Succinate	30	97.6	0.21	97.8	0.35	97.8	0.16
Tatarate	30	91.9	0.185	91.9	0.13	92.6	0.23
Thiourea	50	77	0.31	89.1	0.26	90.2	0.158
Ascorbate	100	97.8	0.22	97.2	0.19	97.2	0.11
SCN^−^	10	85.9	0.17	90.5	0.09	91.1	0.3

*SD standard deviation.

**Table 12:** Effect Interfering ions on the recovery percentage value and limits of tolerance.

Ions	Tolerance limit (mg/L)	Recovery (%)		
		Cu^2+^	Ag^+^	Hg^2+^
Na^+^	1000	99.0	99.3	98.3
K^+^	1000	98.1	98.7	98.2
Mg^2+^	500	97.8	98.4	98.2
Ca^2+^	500	97.8	99.1	99.0
Al^3+^	50	97.5	96.4	95.7
PO_4_^3−^	500	99.0	98.2	99.0
Acetate	50	97.0	98.7	98.8
Oxalate	50	98.1	98.0	97.2
Citrate	50	96.8	95.4	94.6
NO_3_^−^	200	99.2	99.5	99.9
Cl^−^	500	97.8	97.9	98.7
HCO_3_^−^	1000	96.5	98.3	98.0
SO_4_^2−^	1000	99.2	98.0	99.7
Succinate	30	97.6	97.8	97.8
Tatarate	30	91.9	91.9	92.6
Thiourea	50	77	89.1	90.2
Ascorbate	100	97.8	97.2	97.2
SCN^−^	10	85.9	90.5	91.1

#### The selectivity assessment of mixed metal solutions

A mixture of the three studied metals was prepared, to check the effectiveness of DAC@TCH in the adsorption of metal ions in multi-component solutions. The amounts of various metal ions in the studied solutions were measured using ICP OES. In compliance with (Table 13), removal efficiency has no significant variations from that in a single metal ion solution. The findings are consistent with the inference made in the section on interacting ions that shows that DAC@TCH would perform well in multi-component solutions.

**Table 13:** Removal of the studied metal ions (Hg^2+^, Cu^2+^, and Ag^+^) from multicomponent metal ion solution using DAC@TCH.(n = 3).

Sample	Metal ion	Initial concentration (ppm)	Final concentration (ppm)	Removal (%)
Multi-component solution of (Cu^2+^, Hg^2+^, and Ag^+^)	Cu^2+^	20	0.2	99.0
	Hg^2+^	20	0.6	97.0
	Ag^+^	20	0.4	98.00

#### Desorption and reusability studies

Several eluting reagents were investigated for the recovery of adsorbed elements, as shown in (Table 14). The best-used eluent over them was found to be the mixture of 5% thiourea and 0.3 mol / L HNO_3_. 5 mL of the used elution solution was enough volume for complete desorption of the investigated metal ions from the DAC@TCH surface with a recovery percentage over 95.

**Table 14:** Percent recovery for Cu^2+^, Ag^+^ and Hg^2+^ with the eluting solution (n = 3).

Eluting solution	Cu^2+^		Ag^+^		Hg^2+^	
	Recovery, (%)	SD	Recovery, (%)	SD	Recovery, (%)	SD
5% thiourea	85.5	0.84	79.2	0.92	78.2	0.69
0.2 mol/L HNO_3_	75.14	0.63	71.03	0.95	72	0.8
0.3 mol/L HNO_3_	83.01	0.41	80	0.74	75.4	0.94
5% thiourea–0.2 mol/L HNO_3_	91.7	0.71	87.6	0.21	87.9	0.17
5% thiourea–0.3 mol/L HNO_3_	98.9	0.18	96.8	0.34	97.4	0.11

Five successive adsorption–desorption cycles were executed under the investigated metal ions adsorption optimum conditions to assess the DAC@TCH reusability. The findings are shown in (Table 15). After five cycles, DAC@TCH still has about 95 percent of its original capacity for the studied metal ions. This proves that DAC@TCH efficiency didn’t exhibit a significant decrease. So, DAC@TCH is a suitable and promising adsorbent for Cu^2+^, Ag^+^. and Hg^2+^ removal from different real water samples.

**Table 15:** Repeated metal ions (Cu^2+^, Hg^2+^, and Ag^+^) adsorption cycles by using DAC@TCH.

Cycle number	Recovery (%)		
	Cu^2+^	Ag^+^	Hg^2+^
1	99.5	99.8	99.9
2	98.7	98.6	98.5
3	97.6	97.2	97.6
4	96.3	96.6	96.5
5	94.5	95.3	95.8

#### Plausible mechanism of adsorption

The Cu^2+^, Hg^2+^, and Ag^+^ adsorption mechanism onto the DAC@TCH could be explained according to the FTIR and optical images investigation of the DAC@TCH before and after metal ions loading.

##### Optical images

**Fig. 12:**
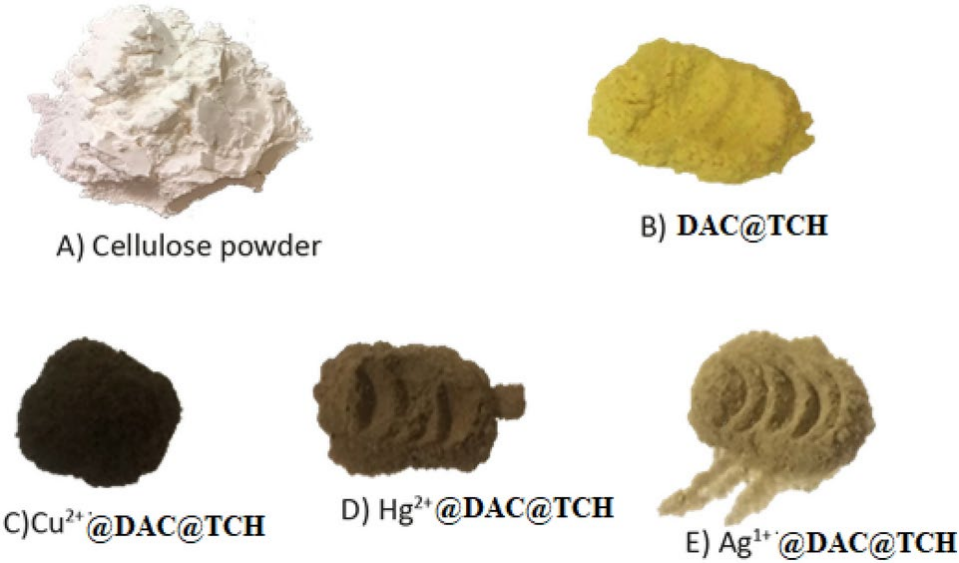
Optical images of (**A**) cellulose, (**B**) DAC@TCH (**C**) DAC@TCH-Cu^2+^ (**D**) DAC@TCH-Hg^2+^and (**E**) DAC@TCH-Ag^+^.

Figure 12A–E displays the optical images of native cellulose, modified one (DAC@TCH) before and after metal-loading (DAC@TCH-Cu^2+^, DAC@TCH- Hg^2+^, and DAC@TCH-Ag^+^), respectively. The photographs showed a clear color difference between the DAC@TCH before and after metal uptake. As the DAC@TCH yellow color (Fig. 12A) converted to dark brown, pale brown, and beige for DAC@TCH-Cu^2+^, DAC@TCH-Hg^2+^, and DAC@TCH-Ag^+^ as shown in (Fig. 12C–E), respectively. These findings demonstrate that the examined metal ions completely penetrated and adsorbed within the modified DAC@TCH^75^.

##### FTIR spectra of DAC@TCH loaded metal ions

**Fig. 13:**
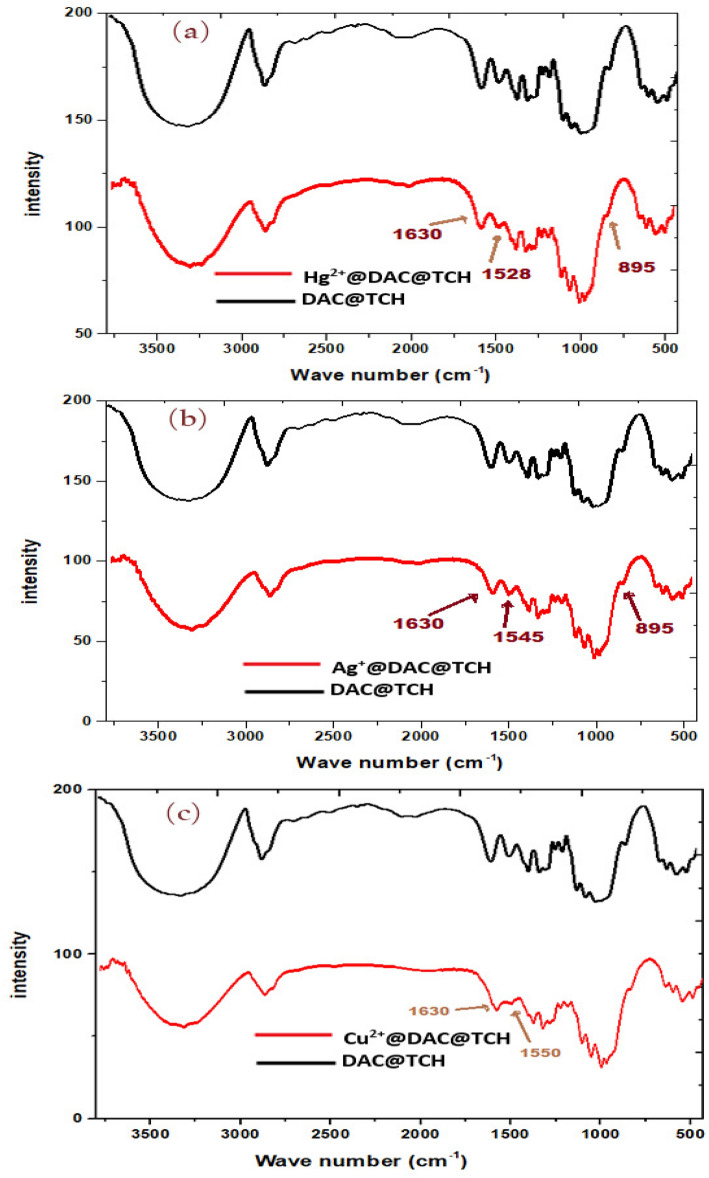
FTIR spectra of DAC@TCH and its complexes with (**a**) DAC@TCH-Hg^2+^, (**b**) DAC@TCH-Ag^+^, and **(c)** DAC@TCH-Cu^2+^.

The FTIR spectrum for the DAC@TCH before and after Cu^2+^, Hg^2+^, and Ag^+^ metal ions adsorption onto its surface was studied to elucidate the adsorption mechanism. The investigated metal ions can coordinate with the inserted thiocarbohydrazide active moieties onto the oxidized cellulose. The findings are represented in (Fig. 13). As predicted, the main distinguishing peaks of thiocarbohydrazide moieties showed obvious changes. As the shift of bending vibrations for the N–H bond from 1540 cm^-1^ to ~ 1528 cm^−1^ for DAC@TCH–Hg^2+^, to ~ 1550 cm^−1^ for DAC@TCH–Cu^2+^, and to ~ 1545 cm^−1^ for DAC@TCH–Ag^+^. Moreover, the shift of the stretching vibrations of the N = C group from 1640 cm^−1^ to ~ 1630 cm^−1^ for the investigated metal ion. Also, the shifting of bending vibrations of the S=C group from 900 to ~ 895 cm^−1^ for three adsorbed metal ions is shown in (Fig. 13a–c).

This study suggests that chemisorption is the dominating mechanism by which the divalent metal ions adhere to DAC@TCH. The following findings support this proposal:

1. As DAC@TCH is rich in N and S functional groups, it can operate as Lewis bases that form stable coordination complexes with Ag^+^, Hg^2+^, and Cu^2+^ metal ions that serve as Lewis acids.
2. According to the data from the isotherm and kinetic section, the DAC@TCH adsorbent followed a pseudo-second-order model, which enhanced the chemisorption in a monolayer manner.
3. From IR results, it can be concluded that the cyano (–C=N), 2ry amine (–NH), C=S, and 1ry amine –NH_2_) groups of the DAC@TCH are responsible for the chelation of the investigated metal ions by DAC@TCH. At pH 6, OH- groups and water molecules can be utilized to complete the coordination number. It is improbable that one substrate of DAC@TCH will be used per metal ion and that’s due to the resulting steric effect from the bonding to the surface of DAC@TCH.

**Fig. 14:**
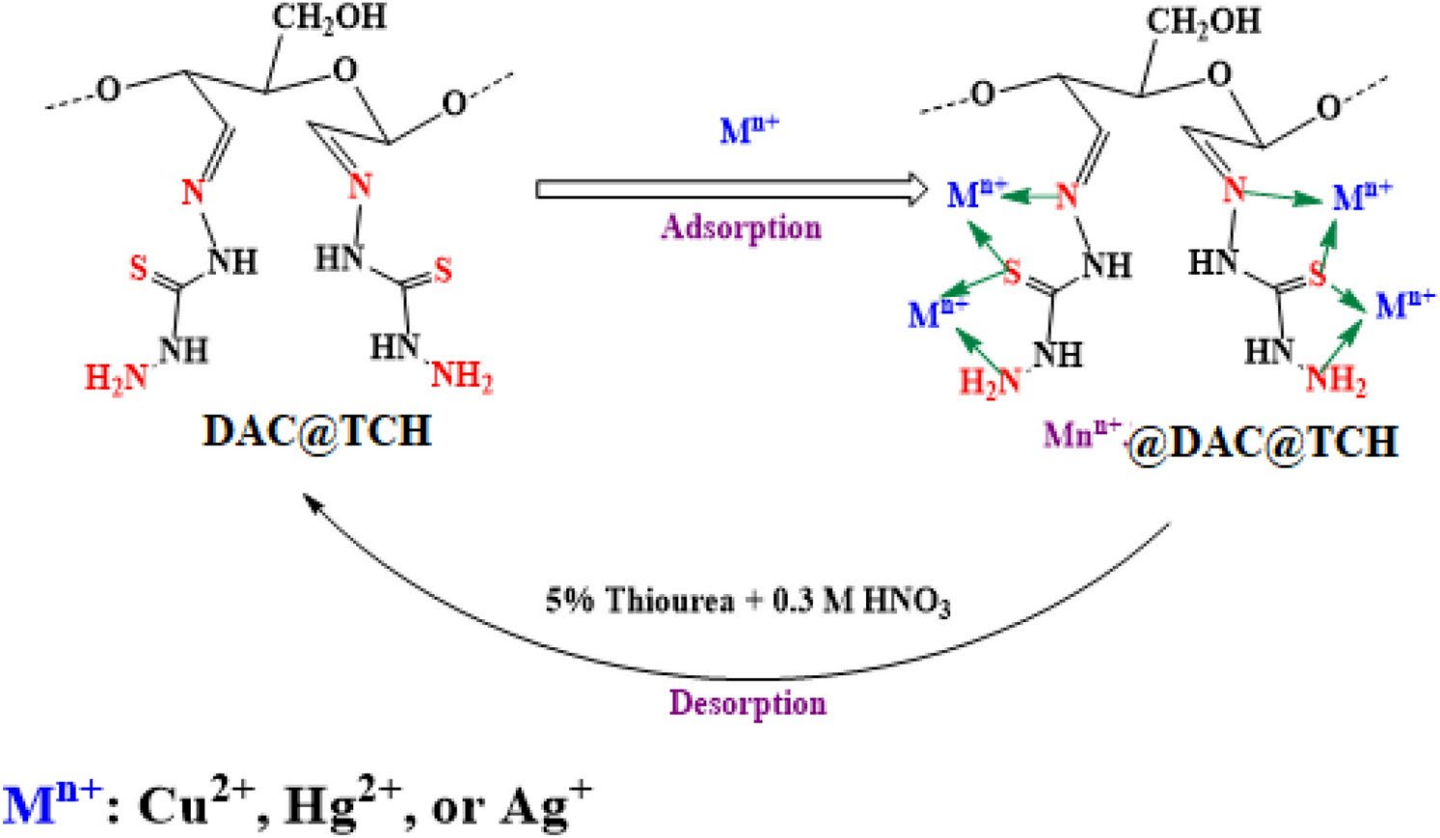
Plausible mechanism of M^n+^ ions adsorption and desorption with DAC@TCH*.*

The presence of so many binding sites in DAC@TCH explains the high affinity for the adsorption of investigated metal ions. Figure 14 shows a hypothetical structure that could be used to explain the mechanism of the adsorption–desorption.

#### Applications

The Cu^2+^, Hg^2+^, and Ag^+^ were recovered from various real samples utilizing the DAC@TCH cellulosic material. The investigated samples, surface water, tap water, and groundwater, were collected from Sinbellawien water station, EL-Mansoura University, and Belbeis Desert, respectively. The results in Table 16 reveal that the prepared DAC@TCH material could be utilized for Cu^2+^, Hg^2+^, and Ag^+^ ions detection in the investigated real samples with high accuracy and precision.

**Table 16:** Analysis of metal ions (Hg^2+^, Cu^2+^, and Ag^+^) in tap, ground, and surface water samples using ICP OES after adsorption by DAC@TCH adsorbent. (Conditions: pH = 6, 500 mg dose, at 298 k, *n* = 3, *ND* not detected).

Sample	Metal ions	Spiked	Measured	Recovered	Recovery, (%)	SD^a^	RSD^b^ (%)
Tap water	Cu^2+^	0.00	0.24	0.24	100	0.002	0.9
		5.00	5.20	5.16	99.2	0.09	1.7
	Hg^2+^	0.00	ND	0.00	0.00	0.00	0.00
		5.00	5.00	5.00	100	0.075	1.5
	Ag^+^	0.00	ND	0.00	0.00	0.00	0.00
		5.00	5.00	100	100	0.09	1.8
Ground water	Cu^2+^	0.00	0.3	0.30	100	0.003	0.9
		5.00	5.23	5.157	98.6	0.067	1.3
	Hg^2+^	0.00	ND	0.00	0.00	0.00	0.00
		5.00	4.98	4.96	99.6	0.065	1.3
	Ag^+^	0.00	ND	0.00	0.00	0.00	0.00
		5.00	4.99	0.998	99.8	0.08	1.6
Surface water	Cu^2+^	0.00	0.28	0.28	100	0.004	1.4
		5.00	5.26	5.24	99.6	0.068	1.3
	Hg^2+^	0.00	ND	0.00	0.00	0.00	0.00
		5.00	4.96	4.92	99.2	0.059	1.2
	Ag^+^	0.00	ND	0.00	0.00	0.00	0.00
		5.00	4.98	4.96	99.60	0.079	1.6

^a^*SD* standard deviation.

^b^*RSD* relative standard deviation.

#### Performance of DAC@TCH adsorbent

A comparative study of the maximum adsorption capacity for Hg^2+^, Cu^2+^, and Ag^+^ metal ions to other adsorbents in the literature was obtained in Table 17 to evaluate the value of the DAC@TCH. It was found that DAC@TCH showed high adsorption capacity for the Hg^2+^, Cu^2+^, and Ag^+^ ions when compared with other mentioned adsorbents. The morphological properties like structure, surface area, and functional groups of each adsorbent are the main reasons for the difference in the Hg^2+^, Cu^2+^, and Ag^+^ ions uptake values. Due to its isoelectric point pH_pzc_, DAC@TCH might be a desirable adsorbent for anionic species. Desorption is a necessary process on the way to adsorbent regeneration. Due to environmental concerns and the requirement for sustainable development, the latter is a crucial factor to consider when estimating the use of any adsorbent for industrial purposes. Column scale and pilot plant investigations can be used in the future to remove cationic and anionic textile dyes from wastewater in the wastewater treatment plant.

**Table 17:** Comparison of Cu^2+^, Hg^2+^, and Ag^+^ sorption capacity between DAC@TCH and newly published sorbents.

Adsorbent	Ions	Q_m_ mg/g	pH	References
Cross-linked magnetic chitosan-phenylthiourea (CSTU) resin	Hg^2+^	135 ± 3	5.0	^76^
PET fibers modified with thiosemicarbazone	Hg^2+^	120.02	5.0	^77^
	Cu^2+^	96.81		
A chitosan–thioglyceraldehyde Schiff’s base cross-linked magnetic resin (CSTG)	Hg^2+^	98 ± 2	5.0	^78^
	Cu^2+^	76 ± 1		
Nano-TiO_2_ was modified with 2-mercaptobenzimidazole	Ag^+^	128.2	3–9	^79^
Modified chitosan resins	Ag^+^	122.04	6	^80^
Cationic wheat straw	Cu^2+^	33.4	4–4.5	^81^
Manganese oxide-modified vermiculite	Ag^+^	69.2	4	^82^
Silica aerogel modified with mercapto (-HS) functional group	Hg^2+^	181	< 7	^83^
	Cu^2+^	51.2		
Valonia tannin resin	Ag^+^	97.08	5	^84^
Chitosan/CNT nanocomposites	Ag^+^	0.393	3	^85^
Expanded perlite	Ag^+^	8.46	6.5	^86^
	Hg^2+^	0.35		
	Cu^2+^	1.95		
Thiocarbohydrazide-modified cellulose	Ag^+^	194	6	Present work
	Hg^2+^	190		
	Cu^2+^	73		

## Conclusion

Herein, we present a simple method to prepare thiocarbohydrazide-modified cellulose nanobiosorbent (DAC@TCH). The DAC@TCH was characterized by various techniques such as elemental analysis, FTIR, SEM, TGA, and TEM. DFT calculations were utilized to verify the molecular structure, analysis of Frontier Molecular orbitals (FMOs), molecular electrostatic potential (MEP) and reactivity descriptor for all phases. In vitro experiments were conducted to evaluate the biological properties of the DAC@TCH nanobiosorbent.These findings revealed that the synthesized DAC@TCH nanobiosorbent has been observed to show effective antibacterial IZD value against *E. Coli* (28 mm) which is superior to the efficacy of standard drug amoxicillin used (5 mm). Furthermore, in silico antibacterial activities (molecular docking) of the DAC@TCH have indicated these to exhibit excellent efficacy with docking score of (**−**7.4237 kcal/mol) and (−7.1325 kcal/mol) for *Staphylococcus aureus and, E. coli,* respectively. Meanwhile the binding energies (best docking scores) in kcal/mol for Amoxicillin are (−5.8090) and (−6.7442) for *Staphylococcus aureus and, E. coli,* respectively. Drug-likeness rules like Lipinski’s, Veber’s and Egan’s were considered for a more comprehensive.

**Fig. 15:**
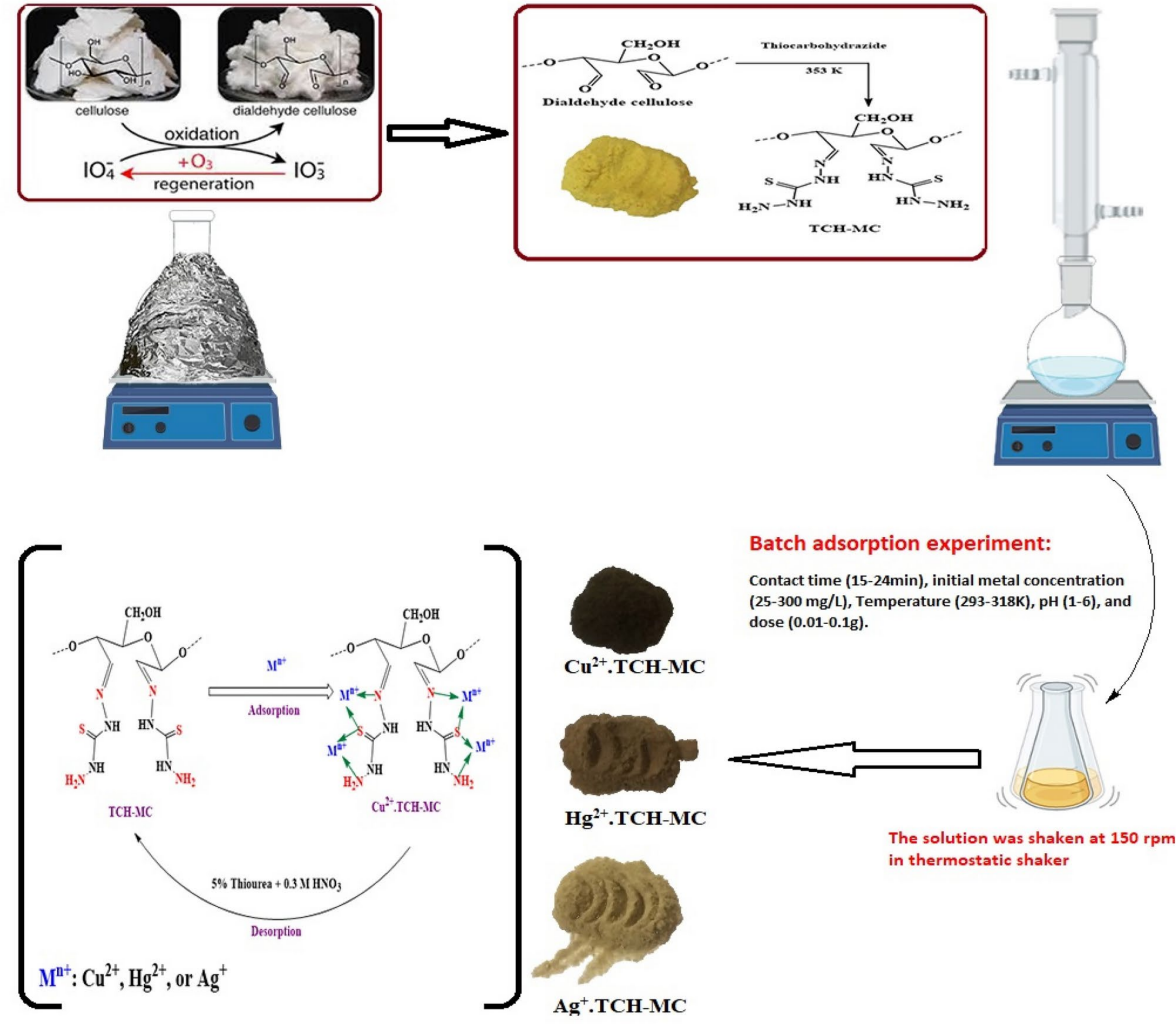
The DAC@TCH synthesis and its use for Hg^2+^, Cu^2+^, and Ag^+^ adsorption and the plausible mechanism of M^n+^ ions adsorption and desorption with DAC@TCH*.*

The prepared DAC@TCH was successfully used for the removal of Cu^2+^, Hg^2+^, and Ag^+^ from aqueous solutions. The optimum parameters like the initial pH, metal initial concentration, contact time, adsorbent dose, and temperature were investigated. When pH is 6, oscillation time is 120 min, and temperature is 25^ο^C, the maximum adsorption capacity was found to be 196 mg/g, 190 mg/g, and 73 mg/g for Ag^+^, Hg^2+^, and Cu^2+^, respectively. The adsorption process was well matched to pseudo-2^nd^-order and Langmuir models. The plausible mechanism of the three metal ions was elucidated. The experimental data proves that DAC@TCH is effective, applicable, and regenerable for metal ions (Cu^2+^, Hg^2+^, and Ag^+^) adsorption from different water sources. The DAC@TCH synthesis and its use for Hg^2+^, Cu^2+^, and Ag^+^ adsorption is shown in (Fig. 15).

## Supplementary Information

Supplementary Information.

## Data Availability

All data generated or analysed during this study are included in this published article [and its supplementary information files].
