# Supplementary material for: Design, spectroscopic analysis, DFT calculations, adsorption evaluation, molecular docking, comprehensive in silico and in vitro bioactivity studies of thiocarbohydrazide grafted dialdehyde cellulose nanobiosorbent
Source: Sci Rep. 2025 Apr 17;15:13319. doi: 10.1038/s41598-025-96525-2 (PMC12006398; doi:10.1038/s41598-025-96525-2)
Supplement: Supplementary file 1 — Supplementary Information. [file 41598_2025_96525_MOESM1_ESM.docx]

**Supplementary data**

**An insight into the adsorptive, computational, molecular docking, and mechanistic behavior of the thiocarbohydrazide grafted dialdehyde cellulose nanobiopolymer in the removal of heavy metal ions**

**Magda A Akl^1*^, Aya G Mostafa^1^, Abdelrahman S Elzeny^1^ and El-Sayed RH El-Gharkawy^1^**

**^1^**Department of Chemistry, Faculty of Science, Mansoura University, Mansoura 35516, Egypt


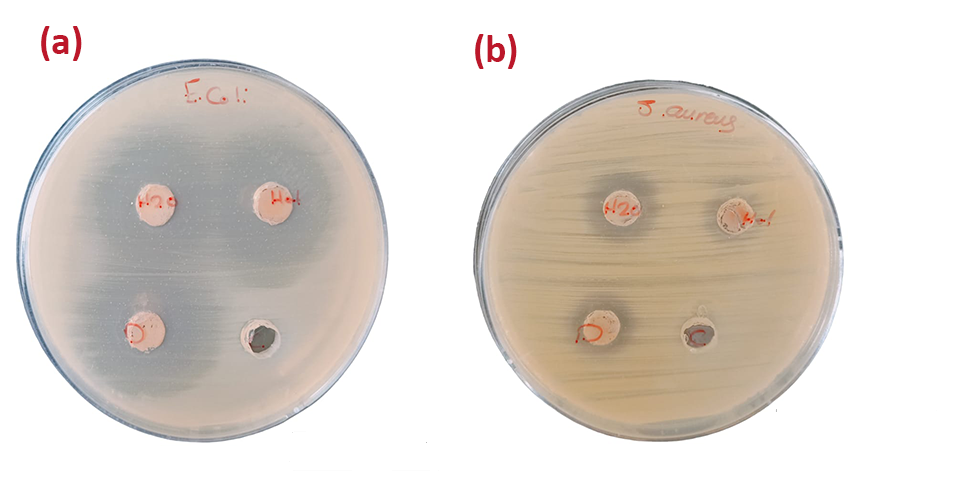


**Figure S1:** **(a)** DAC@TCH to *E. coli* & **(b)** DAC@TCH to *Staphylococcus aureus*

**Figure S2:** Effect of pH on the adsorption performance of DAC@TCH.

(metal ions: 50 ml of 100 mg. L^-1^) DAC@TCH mass: 0.05 g), shaking time: 4 h at 150 rpm, Temp.298 K).

**Figure S3:** Effect of adsorbent dose on adsorption of heavy metals by DAC@TCH

**Figure S4:** Cu^2+,^ Ag^+^, and Hg^2+^ adsorption isotherms by DAC@TCH (50 ml of (25-300) mg/L) initial concentrations, by DAC@TCH sorbent (0.050 g), with rate of shaking 150 rpm for 2 h, pH 6, at 298 K.

|  |  |
| --- | --- |
|  |  |
|  |  |
| **Figure S5:** (a) Langmuir isotherm for Hg^2+^ by DAC@TCH, (b) Freundlich isotherm for Hg^2+^ by DAC@TCH, (c) Langmuir isotherm for Ag^+^ by DAC@TCH, (d) Freundlich isotherm for Ag^+^ by DAC@TCH, (e) Langmuir isotherm for Cu^2+^ by DAC@TCH, and (f) Freundlich isotherm for Cu^2+^ by DAC@TCH. | |

.

**Figure S6:** Effect of oscillation time on the removal efficiency of three single metal ions by DAC@TCH: metal ion (50 ml of 100 mg L^-1^), sorbent (0.05 g), pH 6, rate of shaking 150 rpm at 298 K.

|  |  |
| --- | --- |

**Figure S7:** (a) pseudo-1st-order kinetics for Hg^2+^, Ag+, and Cu^2+^ adsorption on DAC@TCH and (b) pseudo-2nd-order kinetics for Hg^2+^, Ag^+^, and Cu^2+^ adsorption on DAC@TCH.

**
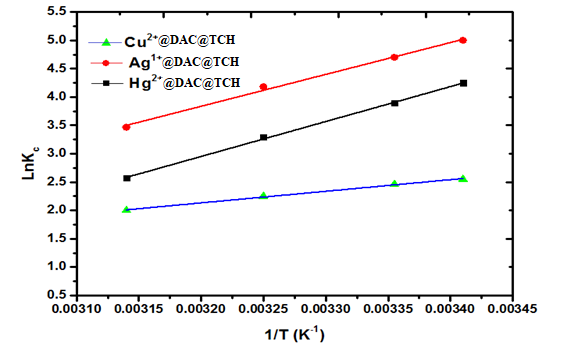
**

**Figure S8**: Plot of ln KC vs 1/T absolute temperature
